# Cohort-Based Reference Values for Serum Ferritin and Transferrin and Longitudinal Determinants of Iron Status in European Children Aged 3–15 Years

**DOI:** 10.1016/j.tjnut.2023.12.001

**Published:** 2023-12-03

**Authors:** Anna Floegel, Timm Intemann, Alfonso Siani, Luis A. Moreno, Dénes Molnár, Toomas Veidebaum, Charalambos Hadjigeorgiou, Stefaan De Henauw, Monica Hunsberger, Gabriele Eiben, Wolfgang Ahrens, Maike Wolters, IDEFICS/I.Family consortia

**Affiliations:** 1 Section of Dietetics, Faculty of Agriculture and Food Sciences, Hochschule Neubrandenburg – University of Applied Sciences, Neubrandenburg, Germany; 2 Leibniz Institute for Prevention Research and Epidemiology – BIPS, Bremen, Germany; 3 Institute of Food Sciences, National Research Council, Avellino, Italy; 4 GENUD (Growth, Exercise, Nutrition and Development) Research Group, Faculty of Health Sciences, Universidad de Zaragoza, Instituto Agroalimentario de Aragón (IA2), Instituto de Investigación Sanitaria Aragón (IIS Aragón), Zaragoza, Spain; 5 Centro de Investigación Biomédica en Red de Fisiopatología de la Obesidad y Nutrición (CIBERObn), Instituto de Salud Carlos III, Madrid, Spain; 6 Department of Pediatrics, Medical School, University of Pécs, Pécs, Hungary; 7 National Institute for Health Development, Estonian Centre of Behavioral and Health Sciences, Tallinn, Estonia; 8 Research and Education Institute of Child Health, Strovolos, Cyprus; 9 Department of Public Health and Primary Care, Ghent University, Ghent, Belgium; 10 Section for Epidemiology and Social Medicine, Department of Public Health and Community Medicine, Institute of Medicine, Sahlgrenska Academy, University of Gothenburg, Gothenburg, Sweden; 11 Department of Public Health, School of Health Sciences, University of Skövde, Skövde, Sweden; 12 Faculty of Mathematics and Computer Science, Institute of Statistics, University of Bremen, Bremen, Germany

**Keywords:** ferritin, transferrin, iron deficiency, reference percentiles, child health

## Abstract

**Background:**

Reference values of ferritin and transferrin for European children do not exist.

**Objective:**

We aimed to provide sex-, age-, and body mass index (BMI)-specific serum ferritin and transferrin reference percentiles of 3–15-y-old children based on cohort data and to investigate determinants of iron status.

**Methods:**

A total of 3390 ferritin and 3416 transferrin measurements from children residing in 8 European countries participating in the IDEFICS/I.Family cohort (https://www.isrctn.com/ISRCTN62310987) at baseline (W0) and 6 y later (W3) were used to estimate percentiles using the generalized additive model for location, scale and shape. Associations of serum ferritin and transferrin concentrations with total iron intake, total iron intake additionally adjusted for vitamin C intake, and iron from heme sources were investigated separately with adjustment for sex, age, country of residence, parental education, usual energy intake and BMI z-score in regression models using cross-sectional and longitudinal data.

**Results:**

The age-specific ferritin and transferrin 5th and 95th reference percentiles ranged from 10.9 to 81.1 μg/L and 2.23 to 3.56 g/L, respectively. A deficient iron status was observed in 3% of children at W0 and 7% of children and adolescents at W3, respectively. At both waves, a higher iron intake from heme sources was positively associated with serum ferritin {W0: β = 3.21 [95% confidence interval (CI): 0.71, 5.71]; W3: β = 4.48 [95% CI: 2.09, 6.87]}, that is, children consuming one mg more heme iron had a 3.21 and 4.48 μg/L higher ferritin concentration. Adherence to a mainly vegetarian diet was associated with a lower chance for sufficient serum ferritin cross-sectionally at W3 [odds ratio (OR) 0.40 (95% CI: 0.21, 0.81)] and longitudinally [OR 0.35 (95% CI: 0.15, 0.93)].

**Conclusions:**

Age-, sex-, and BMI-specific reference percentiles of serum ferritin and transferrin concentrations based on cohort data are provided for European children aged 3–15 y and may be used in clinical practice.

## Introduction

Iron deficiency is one of the most common micronutrient deficiencies worldwide and has detrimental effects on children*.* As well as affecting a large number of children in developing countries, it is one of few micronutrient deficiencies that are also significantly prevalent in industrialized countries [[1], [2], [3]]. Iron is a relevant nutrient particularly for infants older than 6 mo and young children but also for older children, for example, menstruating adolescents or those fed a vegan/vegetarian diet [4]. It is an important component of the hemoglobin of red blood cells and myoglobin of the muscles as well as essential for the cognitive and psychomotor development, and low-iron concentrations bare serious consequences in children [5]. Therefore, preventing the progression of iron deficiency in childhood is especially necessary according to the rapid growth of the brain and the nervous system [6,7]. In addition, iron deficiency is the most significant factor that leads to iron deficiency anemia [1,2]. Even in better economic conditions, as in Europe, iron deficiency is commonly caused by malnutrition with a low-iron diet but also by the increased need in periods of growth and increased losses in adolescent girls [7]. Current dietary guidelines promote plant-based diets as win-win diets for human and planetary health [8]. The European Food Safety Authority (EFSA) states that vegetarians have been reported to have lower iron stores, but were usually above the cut-offs for iron deficiency [9]. The bioavailability of iron from European vegetarian diets is not anymore considered substantially different than from diets containing meat sources by EFSA [9].

Most recent research on iron deficiency in children concentrated on low-and-middle income countries in Africa, South America or Asia. In fact, research in children from the Global North is sparse but important as ferritin reference values vary by ethnicity [10]. Obesity is another factor that impacts serum ferritin concentrations. A recent meta-analysis has shown that obese children more than doubled risk for iron deficiency [11], and putative mechanisms include higher iron requirements of obese children as well as inflammatory processes that inhibit iron absorption [12]. Despite the evidence, ferritin reference values have not yet been reported for obese and nonobese children separately. Furthermore, the iron status and iron deficiencies have been studied most extensively in very young children [13,14]. The prevalence of iron deficiency in children aged 6–36 mo was <5% in Northern and Western Europe but reached ≤50% in Eastern European countries, depending on the country, the age and the socioeconomic status of families [13,15]. However, little is known about iron status of children above 3 y and adolescents.

Most previous studies operated with the WHO deﬁnition for iron deficiency in which serum ferritin was used as a measure of stored body iron [16]. The WHO suggests a value of 12 μg/L as cut-off to diagnose iron deficiency for children below the age of 5 y, and a value of 15 μg/L for children of 5 y or older [16]. Ferritin is not only a biomarker reflecting total body iron stores but also an acute-phase protein that rises during acute and chronic inflammatory processes [17]. A poor iron status can therefore also be detected by other indicators than low plasma or serum ferritin such as decreased hemoglobin, increased transferrin or transferrin receptor or different ratios of the aforementioned [15,18]. To conclude, relevant data on ferritin and transferrin reference values and prevalence of iron deficiency for European children is scarce, particularly for those children above the age of 3 y and for relevant subgroups such as obese and nonobese children.

The aim of this study was therefore to provide serum ferritin and transferrin reference values as marker of iron status of 3–15 y old children from 8 European countries participating in the large IDEFICS/I.Family cohort. For this purpose we used the generalized additive model for location, scale and shape (GAMLSS) [19] and derived sex-, age-, and BMI-specific percentile curves. In addition, we investigated if iron status and iron deficiency are associated with overall dietary iron intake, iron intake from heme sources, mainly vegetarian diet and dietary vitamin C intake using the cross-sectional and longitudinal data of this cohort.

## Methods

The Identification and Prevention of Dietary- and Lifestyle-Induced Health Effects in Children and Infants (IDEFICS) study investigated risk factors of overweight and obesity in young European children. It was followed by the I.Family study to which all IDEFICS children and their families were invited and which aimed to detect determinants of lifestyle behaviors, particularly the role of diet, in the development of the health status during the transition from childhood to adolescence. Thus, the IDEFICS/I.Family cohort represents a pan-European population from 8 European countries (Belgium, Cyprus, Estonia, Germany, Hungary, Italy, Spain and Sweden) which included a total of 16,229 children aged 2–9 y at baseline in 2007/2008 (W0). In the framework of the I.Family study, the children were re-examined in a third follow-up in 2013/2014 (W3) in which also siblings were newly recruited. The same standardized measurement methods and procedures were used during all survey waves. These included physical examinations of the participants, the collection of fasting blood samples if children and parents agreed to it and questionnaires concerning lifestyle behaviors such as dietary intakes. Questionnaires were completed by the parents for children aged 11 y and younger, whereas children aged 12 y and older filled out the questionnaires by themselves. For the detailed assessment of dietary intake a computer-assisted (W0) or web-based (W3) 24-h dietary recall (24HDR) was additionally applied [[20], [21], [22]]. In an interview with a parent, information on chronic diseases of the children and their first-degree relatives as well as drug and supplement use was collected. In this study, W0 and W3 data were used. The design and objectives of the IDEFICS/I.Family studies have been described in more detail elsewhere [23,24].

Ethical approval for the IDEFICS/I.Family studies was obtained from the institutional review boards of all study centers (Ethics Committee of the Gent University Hospital, Belgium; Cyprus National Bioethics Committee, Cyprus; Tallinn Medical Research Ethics Committee, Estonia; Ethic Commission of the University of Bremen, Germany; Medical Research Council, Hungary; Ethics Committee of the Local Health Authority, Avellino, Italy; Ethics Committee for Clinical Research of Aragon, Spain; Regional Ethics Research Board in Gothenburg, Sweden). Written informed consent was given by all parents before their children were included in the studies and additionally by children aged 12 y and older. Younger children provided oral consent for the examinations and sample collection.

### Serum ferritin, transferrin, and C-reactive protein (CRP) concentration

Fasting blood samples were collected by venipuncture in the morning. The collection, processing, shipment and storage of blood samples were conducted according to a quality management system for biological samples [25]. In the study center, serum was immediately separated and shipped to Bremen, Germany, on dry ice where it was stored at -80°C until analysis. Serum ferritin, transferrin and CRP concentrations were measured in a central lab at the Institute of Clinical Chemistry and Laboratory Medicine in Greifswald, Germany. Serum ferritin was measured applying N Latex ferritin which is used for the quantitative determination of ferritin by means of particle enhanced immunonephelometry using BN ProSpec System, Siemens Healthcare Diagnostics Products GmbH, Marburg, Germany. The precision of the assay was 0.5 μg/L for ferritin measurement. According to the laboratory, ferritin values below 3 μg/L had to be excluded because they are too low for an accurate assessment. However, no child was below this threshold at any of the survey waves. Measurement of serum transferrin was performed by immunonephelometry on BN ProSpec System using N Antisera to human transferrin, Siemens Healthcare Diagnostics Products GmbH, Marburg, Germany. The limit of quantitation for transferrin was 0.09 g/L. Transferrin values below a laboratory-given cut-off of 1.4 g/L were excluded (*n =* 3) because they were too low to measure transferrin accurately. The interassay coefficient of variation was 4.49% and 4.74% at median level in the ferritin and transferrin assay, respectively.

The WHO cut-off values for serum ferritin concentrations were used to characterize the iron status of the study participants: children aged <5 y were classified as iron deficient if their serum ferritin was below 12 μg/L, whereas for children of 5 y or older, a value of below 15 μg/L was defined as iron deficiency [16]. Because there is no agreed definition of iron deficiency based on transferrin concentrations, no transferrin cut-offs for deficiency were used.

Serum CRP was measured by electrochemiluminescence technology (Protein Multiplex, Meso Scale Discovery).

### Measurements of determinants of iron status and covariates

Body weight was measured to the nearest 0.1 kg using calibrated Tanita scales (adapted for young children) (Tanita Europe GmbH). Children were in a fasting state and wore only light underwear. A calibrated Seca stadiometer (Seca 225/213 stadiometer) was used to measure height to the nearest 0.1 cm. BMI was calculated as weight (kg) divided by height (m) squared. Sex- and age-specific BMI z-scores were determined based on Cole & Lobstein [26]. Waist circumference was measured to the nearest 0.1 cm with relaxed abdomen in upright position, midway between the lowest rib margin and the iliac crest. Waist-to-height ratios were calculated as waist (m) divided by height (m).

Daily dietary iron intake (μg/d) (separately from all sources and heme iron from meat sources only), vitamin C intake (mg/d) and energy intake (kcal/d) were calculated for each recall based on the 24HDR applying the German food composition table (German Nutrient Data Base, BLS II.3.1) [27].

Children were classified as mainly vegetarian if they replied to the specific question to typically exclude meat, poultry, sausage and fish from their diet in the Food Frequency Questionnaire. This does not necessarily imply that they never consume these products, as some of these participants indicated in other questions also to consume meat products or fish occasionally.

The pubertal status was assessed at W3 in children aged 8 y and older (pre-pubertal or pubertal) and was classified as pubertal in girls if menarche had already occurred (yes compared with no) and if voice alterations had already started or were completed in boys (yes compared with no) based on self-reported age at menarche and voice mutation status [28].

The highest education level of the parents according to the International Standard Classification of Education (ISCED) [29] was grouped in 2 categories: low/medium (ISCED level 0–4) or high level (ISCED level 5 and 6).

### Analysis dataset

From the total sample of 16,229 children at W0 and 9640 children at W3, serum concentrations of ferritin and transferrin were measured in subgroups of children. Based on the available number of children with these data (ferritin: *n =* 3390, transferrin: *n =* 3416), different analysis datasets were selected for references curves estimation, cross-sectional (separately for W0 and W3) and longitudinal analysis. For reference curves estimation we used sex-specific pooled samples of W0 and W3 measurements of ferritin (boys: *n =* 1755; girls: *n =* 1635) and transferrin (boys: *n =* 1760; girls: *n =* 1656) with available measurements of age and BMI. For the cross-sectional analysis at W0: *n =* 883 children and at W3: *n =* 1394 children with complete information on ferritin, transferrin and covariates were included. For the longitudinal analysis, *n =* 812 children with complete information on ferritin, transferrin and covariates at W3 as well as on ferritin and transferrin at W0 were included (Supplemental Figure 1).

### Descriptive analysis of study population

The mean and SD of all continuous variables and the absolute numbers and percentages of all categorical variables were calculated for the entire study population and separately for waves W0 and W3. Furthermore, the mean and SD of ferritin and transferrin were calculated by sex, country, weight status category and ISCED.

### Estimation of usual iron and vitamin C intake

First, we excluded recalls with energy of below 250 kcal as recommended by Tooze et al. [30]. Then, we applied the National Cancer Institute (NCI)-method following the regression calibration approach based on nonlinear mixed effects models [31] to estimate the usual daily energy, iron (from all sources; and separately heme sources only) and vitamin C intake as conditional mean intakes given 24HR-reported intakes and several covariates. This method takes the skewness of the intake distribution as well as effects of weekends and sequence of recalls into account. In addition, it corrects for variance inflation caused by daily variation in the diet, that is, the estimated usual intake distribution is much closer to the long-term average intake distribution as the simple individual average of only a few days [32]. Variance inflation lead to biased effect estimates. The NCI-method has shown to increase the precision of usual intake estimates [31,33]. The usual iron and vitamin C intakes were estimated separately for W0 and W3, and for boys and girls, with age and BMI z-score as covariates. For estimation in W0 and W3, an average of 1.5 and 1.9 recall days were available per child, respectively. This analysis was conducted with the statistical software SAS 9.3.

### Reference curve estimation

We estimated sex-specific reference curves of ferritin and transferrin depending separately on age and BMI z-score using GAMLSS [34]. Different models were fitted to the data. Normal, Box-Cox Cole, Box-Cox *t-* and Box-Cox power exponential distribution with distribution parameters depending on constant, linear or cubic smoothing spline functions of age were considered. The Bayesian Information Criterion and worm plots were applied to assess the goodness of fit of these models and to select a final model. Details regarding model selection were described elsewhere [35]. Supplemental Table 1 lists the final selected models.

### Association analysis

A logistic regression model with the outcome sufficient iron status (yes/no) and the exposures usual iron intake and mainly vegetarian diet (yes/no) adjusted for usual energy intake, age, BMI z-score, sex, country, and ISCED were fitted to the data (main model). Furthermore, for sensitivity analysis we fitted 2 other models with slight changes from the main model: *1*) the main model additionally adjusted for usual vitamin C intake and *2*) the main model with the usual iron intake from heme sources instead of complete iron intake as exposure.

We fitted 2 linear models to investigate the associations of the ferritin and transferrin concentrations as continuous variables with the exposures usual iron intake and a mainly vegetarian diet (yes/no), adjusted for the same variables as in the logistic regression model in accordance with the main model. In addition, we carried out a corresponding sensitivity analysis. This analysis was conducted cross-sectionally at W0 for the whole sample and separately for girls and boys. For the corresponding cross-sectional analysis of W3 data, we used the puberty status as an additional covariate.

For the longitudinal analysis, we used the W3 models adjusted for the corresponding W0 outcome. As first sensitivity analysis, we repeated the whole analysis excluding the children with a CRP value >5 mg/L, which suggests acute inflammation, to account for the fact that ferritin is an acute-phase protein and concentrations rise with inflammation. As second sensitivity analysis, we repeated the W3 and longitudinal analysis excluding the children who reported iron supplementation.

Odds ratio (OR) and β coefficients were estimated and the corresponding 95% confidence intervals (95% CI) were calculated. For this part of the analysis the statistical software R 3.6.2 [36] was used.

The confidence intervals of the β estimates in the models with ferritin as outcome in the cross-sectional and longitudinal analysis were checked using the bootstrap technique to account for additional variation introduced by the usual intake estimation. To check whether model assumptions were violated we derived QQ plots for linear regression models and assessed the dispersion parameter for logistic regression models. The results argue for adequate models.

Pearson correlation coefficients were calculated to show bi-variate linear relationships of ferritin and transferrin concentrations with different dietary and metabolic variables at W3.

## Results

### Descriptive analysis

The descriptive characteristics of the study population for percentile curve estimation stratified by examination wave and sex are presented in Table 1. The mean age of the children across both examination waves was 9.6 y (W0: 6.1 y, W3: 11.9 y). Girls and boys showed mean serum ferritin concentrations of 35 μg/L and 36 μg/L; respectively. The mean serum transferrin concentrations were 2.8 g/L in females and 2.9 g/L in males; respectively. In the longitudinal sample, 3% of the children had a deficient iron status at baseline, whereas 7% had a deficient status at W3. At baseline 1% of the children adhered to a mainly vegetarian diet, this percentage had increased to 5% at W3 (Table 2). Biomarker and dietary variables of the longitudinal samples are shown in Supplemental Table 2. Usual iron intake and iron intake from meat was higher in boys than in girls.

**TABLE 1:** Descriptive characteristics of the IDEFICS/I.Family study population for reference percentiles of serum ferritin and transferrin, stratified by examination wave (W0: 2007/2008; W3:2013/2014) and sex

Variable	W0 (2007/2008) and W3 (2013/2014) pooled					W0 (2007/2008)					W3 (2013/2014)				
	*N*	Q25	Median	Mean	Q75	*N*	Q25	Median	Mean	Q75	*N*	Q25	Median	Mean	Q75
All															
Ferritin (μg /L)	3390	22	31	35	43	1354	20	29	34	41	2036	23	32	36	44
Transferrin (g/L)	3416	2.6	2.8	2.8	3	1360	2.6	2.8	2.8	3	2056	2.6	2.8	2.8	3.1
Age (y)	3417	7.2	9.9	9.62	12.8	1361	4.5	6.4	6.12	7.7	2056	10.4	12.1	11.94	13.6
BMI z-score	3417	−0.4	0.3	0.4	1.1	1361	−0.5	0.1	0.2	0.9	2056	−0.3	0.4	0.5	1.2
CRP^1^ (mg/L)	3183	1.9	2.86	16.52	9.52	1343	1.9	2.86	13.79	9.52	1840	0.94	2.88	18.51	10.14
Boys															
Ferritin (μg/L)	1755	22	30	35.7	42	686	19	27.5	32.85	39	1069	23	32	37.53	44
Transferrin (g/L)	1760	2.6	2.8	2.85	3.1	688	2.6	2.8	2.85	3.1	1072	2.6	2.8	2.85	3.1
Age (y)	1761	7.3	9.9	9.63	12.7	689	4.4	6.3	6.08	7.7	1072	10.4	12.1	11.92	13.6
BMI z-score	1761	−0.43	0.3	0.36	1.07	689	−0.58	0.09	0.15	0.84	1072	−0.3	0.47	0.49	1.25
CRP^1^ (mg/L)	1652	1.9	2.86	15.44	9.52	682	1.9	2.86	12.74	7.62	970	1	2.9	17.34	10.82
Girls															
Ferritin (μg/L)	1635	22	31	35.03	43	668	22	30	34.99	42	967	22	31	35.05	43
Transferrin (g/L)	1656	2.6	2.8	2.82	3	672	2.6	2.8	2.78	3	984	2.6	2.8	2.85	3.1
Age (y)	1656	7.1	9.85	9.6	12.8	672	4.5	6.5	6.16	7.8	984	10.4	12.2	11.95	13.6
BMI z-score	1656	−0.39	0.29	0.35	1.07	672	−0.46	0.15	0.23	0.88	984	−0.31	0.42	0.43	1.19
CRP^1^ (mg/L)	1531	1.9	3.5	17.68	10	661	1.9	3.81	14.88	10.48	870	0.88	2.88	19.8	9.44

Abbreviations: IDEFICS, Identification and Prevention of Dietary- and Lifestyle-Induced Health Effects in Children and Infants; W0, first examination wave; W3, third examination wave.

1 The conversion factor from (mg/L) to unit (μg/mL) is 1; to convert to (mg/dL) divide by 10; to calculate (nmol/L) multiply by factor 9.52.

**TABLE 2:** Descriptive characteristics of the IDEFICS/I.Family study sample for the longitudinal analysis; pooled sample and stratified by sex

	Pooled		Boys		Girls	
	*n*	%	*n*	%	*n*	%
Sex						
Boys	410	50	410	100	0	0
Girls	402	50	0	0	402	100
Iron deficiency^1^						
W0: yes	25	3	20	5	5	1
W3: yes	54	7	22	5	32	8
Mainly vegetarian^2^						
W0: yes	9	1	2	0	7	2
W3: yes	42	5	20	5	22	5
Country						
Italy	58	7	30	7	28	7
Estonia	169	21	83	20	86	21
Cyprus	4	0	2	0	2	0
Belgium	72	9	31	8	41	10
Sweden	98	12	50	12	48	12
Germany	133	16	74	18	59	15
Hungary	131	16	67	16	64	16
Spain	147	18	73	18	74	18
BMI category^3^ (W3)						
Underweight	60	7	30	7	30	7
Normal	574	71	285	70	289	72
Overweight	138	17	72	18	66	16
Obese	40	5	23	6	17	4
ISCED^4^						
Low/middle	369	45	191	47	178	44
High	443	55	219	53	224	56
Pubertal status^5^						
Yes	354	44	199	49	155	39

Abbreviations: IDEFICS, Identification and Prevention of Dietary- and Lifestyle-Induced Health Effects in Children and Infants; W0, first examination wave; W3, third examination wave.

1 Iron deficiency defined according to the WHO: children aged <5 y: serum ferritin <12 μg/L; children of 5 y or older: serum ferritin <15 μg/L.

2 Children were classified as vegetarian if they replied to the specific question to typically exclude meat, poultry, sausage and fish from their diet in the Food Frequency Questionnaire. Some of them however reported later on to occasionally consume meat or fish.

3 According to Cole and Lobstein [26].

4 ISCED, International Standard Classification of Education. The highest educational level of the parents was grouped in 2 categories: low/medium (ISCED level 0–4) or high level (ISCED level 5 and 6).

5 Children were considered pubertal in girls if menarche had already occurred (yes vs. no) and in boys if voice alterations had already started or were completed (yes vs. no) based on self-reported age at menarche and voice mutation status.

### Cohort-based reference curves

**FIGURE 1:**
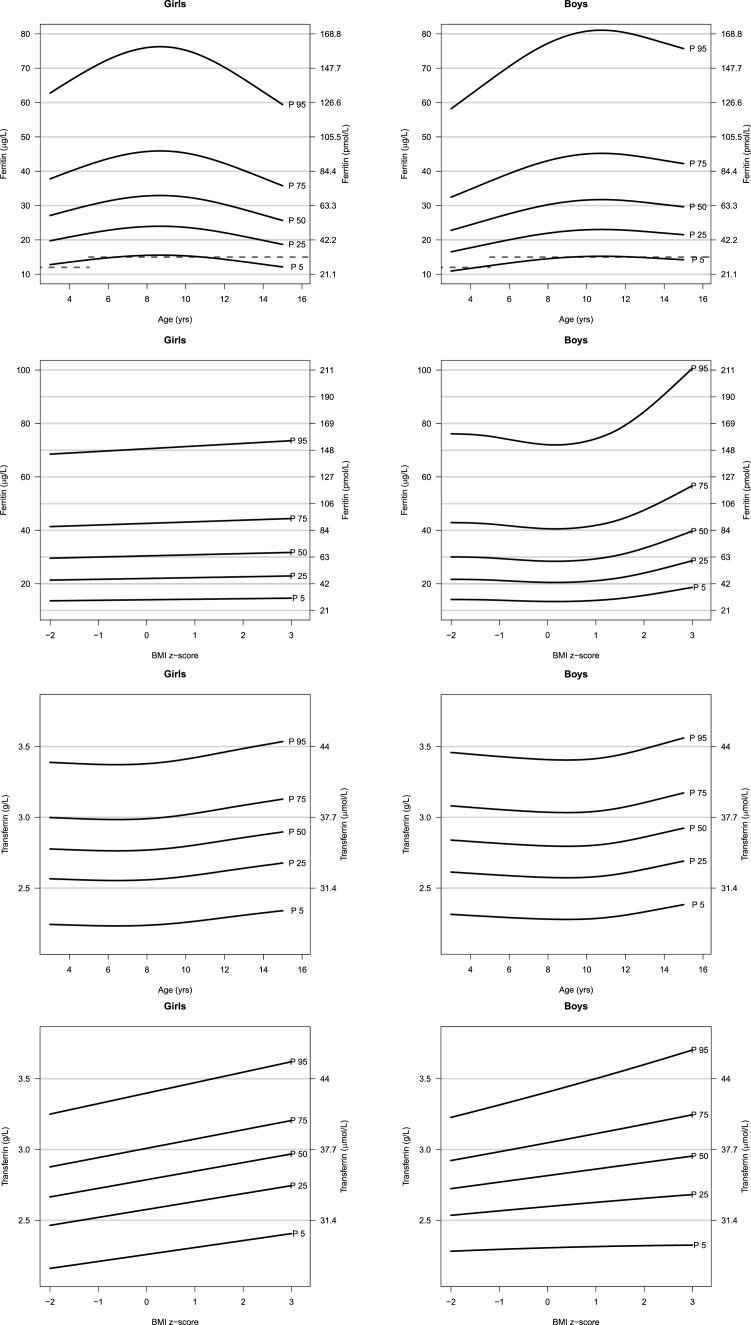
Sex, age-, and BMI-specific reference percentiles of serum ferritin and transferrin concentrations in European children from the IDEFICS/I.Family study aged 3–15 y (study sample: ferritin *n =* 3390; transferrin *n =* 3416). Values below the dotted line indicate iron deficiency as defined by the WHO thresholds for ferritin: 12 μg/L for children <5 y, and 15 μg/L for children ≥5 y [16]. IDEFICS, Identification and Prevention of Dietary- and Lifestyle-Induced Health Effects in Children and Infants.

Figure 1 shows reference percentile curves of serum ferritin and serum transferrin by age and by BMI z-score for girls and boys; respectively. The age-specific 5th and 95th ferritin and transferrin reference percentiles ranged from 10.9 to 81.1 μg/L and 2.23 to 3.56 g/L, respectively. In girls, from 3 to 9.2 y of age, serum ferritin concentrations rise up, whereas in boys, serum ferritin values raise age ≤11.4 y (Supplemental Table 3). Above these ages, serum ferritin concentrations decrease in both sexes. A stronger decrease is observed for girls, which results in lower serum ferritin concentrations at age 15 y compared with age 3 y for females. Serum ferritin concentrations slightly increase with BMI z-scores in girls (Figure 1, Supplemental Table 4). In boys, serum ferritin concentrations slightly decrease for BMI z-scores <0, and above this cut-off strongly increase ≤40 μg/L for the 50th percentile at a BMI z-score of 3.0. Serum transferrin concentrations slightly decrease for the 50th percentile from age 3 y to age 7.3 y in girls, and to age 10.5 in boys; respectively (Figure 1, Supplemental Table 5). Above these ages the 50th percentiles of serum transferrin concentrations increase in both sexes. With increasing BMI z-score, serum transferrin concentrations steadily increase in both sexes (Figure 1, Supplemental Table 6).

### Association analysis

Supplemental Figure 2 shows the correlation coefficients of serum ferritin and transferrin concentrations with dietary and metabolic variables at W3 and with serum ferritin and transferrin concentrations at W0.

### Iron status and serum ferritin concentration

Table 3 shows the results of the logistic and linear regression models adjusted for usual energy intake, age, BMI z-score, sex, country, and educational level. At baseline (W0), the usual iron intake from meat sources (mg/day) was positively associated with ferritin (μg/L) [β = 3.21, 95% CI: (0.71, 5.71)] (Table 3). This association was particularly strong in girls [β = 9.68, 95% CI: (3.10, 16.26)] indicating that girls consuming one milligram more iron from meat products than other girls had a 9.68 μg/L higher ferritin concentration. In addition, total usual iron intake was positively associated with ferritin in the model adjusted for vitamin C in girls (Supplemental Table 7).

**TABLE 3:** Cross-sectional associations of usual total iron intake with serum ferritin (μg/L) and sensitivity analyses in the IDEFICS/I.Family study

Model	Main model			Sensitivity analysis including usual vitamin C intake^2^			Sensitivity analysis with usual iron intake from heme sources^2^ as exposure		
Wave 0 (*N* = 883)									
Logistic regression									
Odds ratio for having a sufficient iron status (ref.: deficient status)^1^	OR	95% CI		OR	95% CI		OR	95% CI	
Total iron intake^2^ (mg/d)	1.36	0.91	2.08	1.35	0.88	2.15	1.28	0.77	2.13
Mainly vegetarian diet^3^ (ref: omnivore diet)	0.44	0.07	8.40	0.44	0.07	8.42	0.48	0.08	9.27
Vitamin C intake^2^ (mg/d)				1.00	0.98	1.03			
Linear model									
β estimate	β	95% CI		β	95% CI		β	95% CI	
Total iron intake^2^ (mg/d)	1.18	−0.50	2.85	0.95	−0.88	2.78	3.21	0.71	5.71
Mainly vegetarian diet^3^	−4.34	−15.55	6.86	−4.24	−15.45	6.98	−2.56	−13.83	8.72
Vitamin C intake^2^ (mg/d)				0.03	−0.06	0.12			
Wave 3 (*N* = 1394)									
Logistic regression									
Odds ratio for having a sufficient iron status (ref.: deficient status)^1^	OR	95% CI		OR	95% CI		OR	95% CI	
Total iron intake^2^ (mg/d)	1.09	0.92	1.31	1.10	0.92	1.33	1.93	1.14	3.39
Mainly vegetarian diet^3^ (ref: omnivore diet)	0.40	0.21	0.81	0.40	0.21	0.81	0.64	0.30	1.41
Vitamin C intake^2^ (mg/d)				1.00	0.98	1.01			
Linear model									
β estimate	β	95% CI		β	95% CI		β	95% CI	
Total iron intake^2^ (mg/d)	0.50	−0.36	1.36	0.25	−0.66	1.16	4.48	2.09	6.87
Mainly vegetarian diet^3^ (ref: omnivore diet)	−3.75	−8.16	0.66	−3.72	−8.13	0.68	−0.27	−5.03	4,49
Vitamin C intake^2^ (mg/d)				0.06	−0.01	0.13			

Abbreviations: β, β estimate; CI, confidence interval; IDEFICS, Identification and Prevention of Dietary- and Lifestyle-Induced Health Effects in Children and Infants; OR, odds ratio.

Adjusted for usual energy intake, age, BMI-z-score, sex, country and ISCED.

1 Iron deficiency defined according to the WHO: children aged <5 y: serum ferritin <12 μg/L; children of 5 y or older: serum ferritin <15 μg/L.

2 Usual dietary intakes of total iron, iron from heme sources and vitamin C were estimated from 24-h dietary recalls linked to food composition tables.

3 Children were classified as mainly vegetarian if they replied to the specific question to typically exclude meat, poultry, sausage and fish from their diet in the food frequency questionnaire. Some of them reported to occasionally consume meat or fish.

At W3, the usual iron intake from meat sources was positively associated with a sufficient serum ferritin status [OR 1.93 (95% CI: 1.14, 3.39)] indicating that children consuming one additional milligram iron from meat sources had almost twice the chance of a sufficient than a deficient iron status. In contrast, adherence to a mainly vegetarian diet, also if additionally adjusted for vitamin C, was associated with a 60% lower chance of sufficient serum ferritin [OR 0.40 (95% CI: 0.21, 0.81)] compared with an omnivore diet (Table 3). Prevalence of iron deficiency among mainly vegetarians at W3 (*N =* 75) was 16% compared with 7% among omnivores (*N =* 1319). At W3, the linear model shows that usual iron intake from meat sources was again positively associated with serum ferritin concentrations [β = 4.48 (95% CI: 2.09, 6.87)] which means that children consuming one milligram more iron from meat products than other children had a 4.48-μg/L higher ferritin concentration (Table 3). A slightly stronger association was observed in the subgroup of boys than in girls (Supplemental Table 7).

Longitudinally, a mainly vegetarian diet at W0 was associated with a 65% lower chance of sufficient serum ferritin at W3 [OR 0.35 (95% CI: 0.15, 0.93)], both in the main model and after further adjustment for vitamin C (Table 4). In the linear model, iron intake from meat sources was longitudinally positively associated with serum ferritin [β = 3.64 (95% CI: 0.93, 6.35)] indicating that children consuming one milligram more iron from meat sources than others with the same ferritin concentration at W0 had a 3.64 μg/L higher ferritin concentration at W3 (Table 4). Supplemental Table 8 shows the longitudinal associations stratified by sex.

**TABLE 4:** Longitudinal associations of usual total iron intake with serum ferritin (μg/L) at W3 (2013/2014) adjusted for serum ferritin at W0 (2007/2008) and sensitivity analyses in the IDEFICS/I.Family study

Model	Main model			Sensitivity analysis including usual vitamin C intake^2^			Sensitivity analysis with usual iron intake from heme sources^2^ as exposure		
*N =* 810									
Logistic regression									
Odds ratio for having a sufficient iron status at W3 (ref.: deficient status)^1^	OR	95% CI		OR	95% CI		OR	95% CI	
Total iron intake^2^ (mg/d)	1.07	0.86	1.36	1.10	0.87	1.42	2.04	0.98	4.55
Mainly vegetarian diet^3^ (ref: omnivore diet)	0.35	0.15	0.93	0.35	0.15	0.93	0.59	0.22	1.78
Vitamin C intake^2^ (mg/d)				1.00	0.98	1.02			
Linear model									
β estimate	β	95% CI		β	95% CI		β	95% CI	
Total iron intake^2^ (mg/d)	−0.01	−0.95	0.92	0.01	−1.00	1.01	3.64	0.93	6.35
Mainly vegetarian diet^3^ (ref: omnivore diet)	−4.61	−9.87	0.64	−4.61	−9.87	0.65	−1.7	−7.36	3.97
Vitamin C intake^2^ (mg/d)				0	−0.09	0.08			

Abbreviations: β, β estimate; CI, confidence interval; IDEFICS: Identification and Prevention of Dietary- and Lifestyle-Induced Health Effects in Children and Infants; OR, odds ratio; W0, first examination wave; W3, third examination wave.

Adjusted for usual energy intake, age, BMI-z-score, sex, country, ISCED (all at W3) and serum ferritin at W0.

1 Iron deficiency defined according to the WHO: children aged <5 y: serum ferritin <12 μg/L; children of 5 y or older: serum ferritin <15 μg/L.

2 Usual dietary intakes of total iron, iron from heme sources and vitamin C were estimated from 24-h dietary recalls linked to food composition tables.

3 Children were classified as mainly vegetarian if they replied to the specific question to typically exclude meat, poultry, sausage and fish from their diet in the food frequency questionnaire. Some of them however reported later on to occasionally consume meat or fish.

### Serum transferrin concentration

Table 5 shows the cross-sectional and longitudinal associations for transferrin. At baseline, a positive association of a mainly vegetarian diet with transferrin was detected in boys [β = 0.33 (95% CI: 0.05, 0.61)] indicating a 0.33 g/L higher serum transferrin concentration if participants typically excluded meat, poultry, sausage and fish from their diet (Supplemental Table 9). However, this was based on only 22 boys adhering to a mainly vegetarian diet at W0 or W3 (see Table 2).

**TABLE 5:** Cross-sectional and longitudinal associations (linear model) of usual total iron intake with serum transferrin (g/L) and sensitivity analyses in the IDEFICS/I.Family study

Model	Main model			Sensitivity analysis including usual vitamin C intake^1^			Sensitivity analysis with usual iron intake from heme sources^1^ as exposure		
Wave 0 (*N =* 833)									
β estimate	β	95% CI		β	95% CI		β	95% CI	
Total iron intake^1^ (mg/d)	0	−0.03	0.03	0.01	−0.02	0.05	−0.02	−0.07	0.02
Mainly vegetarian diet^2^ (ref: omnivore diet)	0.16	−0.05	0.36	0.15	−0.05	0.35	0.14	−0.06	0.35
Vitamin C intake^1^ (mg/d)				0	−0.003	0.0001			
Wave 3 (*N* = 1394)									
β estimate	β	95% CI		β	95% CI		β	95% CI	
Total iron intake^1^ (mg/d)	0	−0.01	0.02	0	−0.02	0.02	−0.01	−0.06	0.03
Mainly vegetarian diet^2^ (ref: omnivore diet)	0.06	−0.02	0.14	0.06	−0.02	0.14	0.05	−0.04	0.14
Vitamin C intake^1^ (mg/d)				0	−0.001	0.002			
Longitudinal (*N* = 812) (i.e., at W3 adjusted for serum transferrin at W0)									
β estimate	β	95% CI		β	95% CI		β	95% CI	
Total iron intake^1^ (mg/d)	0	−0.01	0.02	0	−0.02	0.02	0.02	−0.03	0.06
Mainly vegetarian diet^2^ (ref: omnivore diet)	0.05	−0.04	0.14	0.05	−0.04	0.14	0.07	−0.03	0.17
Vitamin C intake^1^ (mg/d)				0	−0.001	0.002			

Abbreviations: β, β estimate; CI, confidence interval; IDEFICS: Identification and Prevention of Dietary- and Lifestyle-Induced Health Effects in Children and Infants; W0, first examination wave; W3, third examination wave.

Adjusted for usual energy intake, age, BMI-z-score, sex, country and ISCED.

1 Usual dietary intakes of total iron, iron from heme sources and vitamin C were estimated from 24-h dietary recalls linked to food composition tables.

2 Children were classified as mainly vegetarian if they replied to the specific question to typically exclude meat, poultry, sausage and fish from their diet in the food frequency questionnaire. Some of them however reported later on to occasionally consume meat or fish.

### Sensitivity analyses

The sensitivity analysis excluding children with increased CRP concentrations (higher than 5 mg/L) led to similar results (Supplemental Tables 10–12). In addition, a sensitivity analysis excluding children who used iron supplements did not markedly change the results.

## Discussion

Our cohort-based study in European children and adolescents aged 3–15 y provides sex-specific ferritin and transferrin percentile curves by age and BMI z-score based on 3390 and 3416 observations, respectively. These data can be informative for pediatricians and researchers to classify the iron status of individual children and adolescents not only based on the WHO ferritin cut-offs to define deficiency but also considering age- and sex-specific percentiles for ferritin and transferrin.

Further, our study evaluates the impact of usual dietary iron intake, a mainly vegetarian diet, usual vitamin C intake and usual iron intake from meat sources on serum ferritin and transferrin concentrations. To our knowledge this is the first study that provides pan-European ferritin and transferrin reference percentiles for children and adolescents. Previous studies reported national reference percentiles [[37], [38], [39], [40]] or reference percentiles for specific age groups or limited age ranges only [41].

In line with previous results from an Australian study [37], serum ferritin concentrations increased in girls age ≤9 and in boys age ≤11 to values around 30 μg/L (50th percentile) and decreased afterwards in our population whereas an additional increase from the age of 14 was observed in Australian [37] and Canadian boys [42]. At young ages, boys had lower ferritin values than girls whereas from the age of 10–11 y girls had lower values and a stronger decrease than boys as also reported from 2 Australian studies [37,43]. The lower values in post-menarche girls may be due to menstrual iron losses as indicated by previous studies [44] and by a lower iron intake of girls compared with boys which was also shown previously [45,46]. Menstrual losses have a strong effect as shown in a Greek study which reported the iron status among 9–13-y old girls. The prevalence of iron deficiency was 33.5% in girls with menses compared with 15.9% in girls without menses [44]. In accordance with a small study in Sweden based on a local sample [47], we observed higher transferrin concentrations during adolescence compared with the concentrations in younger childhood but in contrast to the Swedish children, adolescent girls did not have higher values in the teens than boys.

At baseline, in our study the prevalence of iron deficiency was higher in boys than in girls (5% compared with 1%) whereas at 6-y-follow-up the prevalence was lower in boys than in girls (5% compared with 8%). In a Norwegian sample of 6–12-y-old children, the prevalence of iron deficiency as well as mean serum ferritin and transferrin were similar to our study [39]. A United States study reported highest prevalence of iron deficiency in 12–16-y-old children (4.7%) compared with younger age groups [48]. We also observed the highest prevalence of deficiency in the oldest age groups, mainly in 11–15-y-old girls. Thus, the lower ferritin reference limits, that is, the 5th percentile references, for this age group, were below the WHO cut-off for iron deficiency.

Some previous studies indicated that children and adults with overweight or obesity seem to be at increased risk of iron deficiency [48,49]. In our study, slightly rising transferrin concentrations with increasing BMI may reflect a similar trend although this is not supported by the slightly increasing ferritin concentrations with higher BMI z-scores, and higher ferritin concentrations particularly in obese boys. To the contrary, excess of visceral fat can trigger a chronic inflammatory state which can elevate ferritin transcription via pro-inflammatory cytokines resulting in higher ferritin concentrations [49,50]. Accordingly, several studies reported high ferritin concentrations despite a low-iron status [49,51]. However, it is not yet clear whether an excess weight status actually leads to iron deficiency as previous studies in adolescents did not report an impaired iron status or higher prevalence of iron deficiency in those classified as overweight or obese compared with those with normal weight [52,53]. We tried to rule out the effects of inflammation on ferritin concentrations by excluding children with high CRP concentrations which revealed similar results.

In our association analyses, iron intake from heme sources was most strongly associated with serum ferritin in all models and more important for iron status than total usual iron intake. In fact, an additional intake of one milligram iron from heme sources almost doubled the chance of a non-deficient status. Previous studies have also shown the crucial role of heme iron for iron status. In a Canadian study traditional meat intake was positively associated with serum ferritin in 3–19-y-old children and adolescents [54]. In Swedish girls aged 17 y, the frequency of meat consumption was the only predictor for serum ferritin [45].

Adhering to a mainly vegetarian diet was inversely associated with serum ferritin concentrations in our study. However, these results must be interpreted with caution because the number of children with this kind of diet was small. A recent review found that most but not all studies reported a higher prevalence of iron deficiency in children and adolescents consuming a vegetarian diet [55]. Other studies reported no effect of a vegetarian diet on the iron status of children and adolescents [56,57]. However, in the latter study [56], the participants were 5 to 10 y old, therefore, menstrual losses may have not yet been relevant.

The results of our investigation regarding effects of vitamin C seem to be contradictory to other studies. As vitamin C can increase the absorption of non-heme iron, adjustment of the models for vitamin C may increase the associations with serum ferritin or transferrin. In a Canadian study, fruit, and fruit juice because of their vitamin C content were positively associated with serum ferritin [54]. Nevertheless, in our models, adjustment for vitamin C had neither an effect of its own nor did it modify the effects of a mainly vegetarian diet or that of usual iron intake. Please note that we only reported 95% CI without adjusting for multiple testing to report precision of estimates and to ensure comparability. Therefore, interpretation focus only on effect estimates and not on statistical significance.

Considering the adverse effects of a high consumption of meat and meat products on health outcomes such as cardiovascular diseases and colorectal cancer as well as the climate impact of meat production due to high concentrations of greenhouse gas emissions, there is growing consensus among nutrition/medical societies and climate researchers to recommend plant-based diets with low proportions of meat [8,58,59]. Based on these facts and for animal welfare, an increasing number of young people adhere to vegetarian and vegan diets and should be aware of potential deficits of iron and other micronutrients.

### Strengths and limitations

Our study had several limitations. The information on iron supplement use was only collected at follow-up in our study. However, this concerned only very few children in our analytical dataset (*n =* 7 of 1394 in W3). A sensitivity analysis revealed that the results did not markedly change when we excluded these children for the association analysis. The pubertal status was assessed only at follow-up in children aged 8 y and older and not at baseline when children were 2–9 y old. Thus, we may have missed a few children who were already pubertal at baseline below the age of 10 y. Furthermore, we only had a small number of children and adolescents with a mainly vegetarian diet. Thus, results for mainly vegetarian diets have to be interpreted with caution as they are based on a small number of children. In addition, our population is not representative for European children but may be considered as an unselected population of Caucasian children and adolescents compared with clinical populations which are often used for the calculation of reference values [42,60]. For pediatricians, our percentiles can add important information to the commonly used WHO definition for deficiency based on ferritin as further information on how to evaluate serum ferritin and transferrin values of patients in the context of age-, sex-, and BMI-specific percentiles from of mainly healthy children is provided. Nevertheless, the calculated percentiles do not necessarily reflect optimal concentrations and may have been biased if the willingness to participate in the study is associated with the iron status. In this regard, the socioeconomic status can be an essential factor which is probably associated with both.

An important strength of our study was that percentile curves of serum ferritin and transferrin concentrations were calculated by GAMLSS based on a large population of children and adolescents from different regions across Europe. Another strength is that the design of our study allowed cross-sectional and longitudinal association analysis considering multiple determinants of iron status at baseline and after 6 y of follow-up. Participants of this cohort study were extensively phenotyped including blood sampling. Ferritin and transferrin concentrations were analyzed in a central laboratory. Study teams across countries adhered to highly standardized protocols and standard operating procedures for all examinations.

## Conclusion

The current study provides age-, sex-, and BMI-specific ferritin and transferrin reference percentiles based on a large cohort of European children and adolescents for the clinical and scientific community. Such data is currently missing and can be used by pediatricians to classify the iron status of individual children and adolescents not only based on the WHO definition for deficiency but also considering age-, sex-, and BMI-specific percentiles. Dietary iron intake from heme sources but not total dietary iron intake was found as a determinant for sufficient iron status defined by WHO cut-offs of serum ferritin in European children and adolescents. The impact of mainly vegetarian diet on iron status in children and adolescents should be addressed in future studies with higher statistical power, for example, in specific cohorts targeting vegetarians and vegans.

## Acknowledgments

This study includes data from the IDEFICS study and the consequent I.Family study and is published on behalf of both European Consortia. We are very grateful to all the children, adolescents, and parents participating in the IDEFICS study and/or I.Family study. We also wish to thank the school boards, head teachers, and communities for their support and the study nurses, interviewers, laboratory technicians, and data managers for their effort.

## Author contributions

The authors’ responsibilities were as follows – All authors: saw and approved the contents of the submitted manuscript; AF, TI, AS, LAM, DM, TV, CH, SDH, MH, GE, WA, MW: contributed to conception and design, acquisition of data, analysis, or interpretation of data; MW, AF: wrote the original draft; TI: conducted the formal analysis; all authors: approved the final version to be published; and all authors: revised the article critically for important intellectual content.

## Funding

The IDEFICS study (http://www.idefics.eu) was supported by the European Commission within the Sixth RTD Framework Program [Contract No. 016181 (FOOD)] and the I.Family study (http://www.ifamilystudy.eu) was funded within the Seventh RTD Framework Program [Contract No. 266044].

## Data availability

Because of the highly sensitive data collected in children, ethical restrictions prohibit the authors from making the minimal data set publicly available. Data are however available from the authors upon reasonable request and with permission of the Steering Committee on a case-by-case basis. Interested researchers can contact the study co-ordinator (ahrens@leibniz-bips.de) to discuss possibilities for data access.

## Conflict of interest

The authors declare no conflicts of interest.

## Appendix A Supplementary data

Multimedia component1 Multimedia component1
