# Supplementary material for: Cohort-Based Reference Values for Serum Ferritin and Transferrin and Longitudinal Determinants of Iron Status in European Children Aged 3–15 Years
Source: J Nutr. 2023 Dec 3;154(2):658–69. doi: 10.1016/j.tjnut.2023.12.001 (PMC10900138; doi:10.1016/j.tjnut.2023.12.001)
Supplement: Multimedia component1 [file mmc1.pdf]

Supplementary Material

Cohort-based reference values for serum ferritin and transferrin and longitudinal determinants of iron status in European children aged 3 to 15 years

Anna Floegel[1,2*], Timm Intemann[2*], Alfonso Siani[3], Luis A Moreno[4,5], Dénes Molnár[6], Tomaas Veidebaum[7], Charalambos Hadjigeorgiou[8], Stefaan De Henauw[9], Monica Hunsberger[10], Gabriele Eiben[11], Wolfgang Ahrens[2,12], Maike Wolters[2] on behalf of the IDEFICS/I.Family consortia

[1] Section of Dietetics, Faculty of Agriculture and Food Sciences, Hochschule Neubrandenburg - University of Applied Sciences, 17033 Neubrandenburg, Germany

[2]Leibniz Institute for Prevention Research and Epidemiology - BIPS, 28359 Bremen, Germany

[3]Institute of Food Sciences, National Research Council, 83100 Avellino, Italy

[4]GENUD (Growth, Exercise, Nutrition and Development) Research Group, Faculty of Health Sciences, Universidad de Zaragoza, Instituto Agroalimentario de Aragón (IA2), Instituto de Investigación Sanitaria Aragón (IIS Aragón), 50009 Zaragoza, Spain

[5]Centro de Investigación Biomédica en Red de Fisiopatología de la Obesidad y Nutrición (CIBERObn), Instituto de Salud Carlos III, 28029 Madrid, Spain

[6]Department of Pediatrics, Medical School, University of Pécs, 7623 Pécs, Hungary

[7]National Institute for Health Development, Estonian Centre of Behavioral and Health Sciences, 11619 Tallinn, Estonia

[8]Research and Education Institute of Child Health, 2035 Strovolos, Cyprus

[9]Department of Public Health and Primary Care, Ghent University, 9000 Ghent, Belgium

[10]Section for Epidemiology and Social Medicine, Department of Public Health and Community Medicine, Institute of Medicine, Sahlgrenska Academy, University of Gothenburg, 40530 Gothenburg, Sweden.

[11]Department of Public Health, School of Health Sciences, University of Skövde, Skövde, Sweden

[12]Institute of Statistics, Faculty of Mathematics and Computer Science, University of Bremen, 28359 Bremen, Germany

*These authors contributed equally to this work.

Corresponding author

Dr. Maike Wolters
Leibniz Institute for Prevention Research and Epidemiology - BIPS
Achterstr. 30, 28359 Bremen, Germany
Email: wolters@leibniz-bips.de; sec-epi@leibniz-bips.de
Phone: +49 (0)421 218 56845

Supplementary Table 1: Final selected GAMLSS models for estimation of serum ferritin and transferrin percentile curves

Sex	Covariates	Distribution	μ	log(σ)	ν	log(τ)
**Ferritin**						
Male<br>Female	Age	BCCG<br>BCCG	cs(df = 1)<br>cs(df = 1)	const.<br>const.	const.<br>const.	-<br>-
Male<br>Female	BMI-z-score	BCCG<br>BCCG	cs(df = 1)<br>linear	const.<br>const.	const.<br>const.	-<br>-
**Transferrin**						
Male<br>Female	Age	BCCG<br>BCT	cs(df = 1)<br>cs(df = 1)	const.<br>const.	const.<br>const.	-<br>const.
Male<br>Female	BMI-z-score	BCCG<br>BCT	linear<br>linear	linear<br>const.	const.<br>const.	-<br>const.

BCT: Box-Coc-t; BCCG: Box-Cox-Cole-and-Green; cs: cubic smoothing spline; df: additional degree of freedom

Supplementary Table 2: Descriptive characteristics of the IDEFICS/I.Family study sample for the longitudinal analysis; pooled sample and stratified by sex (continuous variables)

	W0 (2007-2008)					W3 (2013/2014)				
	N	Q25	Median	Mean	Q75	N	Q25	Median	Mean	Q75
All:										
Ferritin [µg/L]	810	21.0	29.5	34.3	42.0	812	23.0	33	36.4	44.0
Transferrin [g/L]	812	2.60	2.8	2.81	3.00	812	2.60	2.8	2.85	3.10
Age [years]	812	4.50	6.4	6.12	7.70	812	10.30	12	11.92	13.60
BMI z-score*	812	-0.56	0.1	0.18	0.85	812	-0.34	0.38	0.41	1.16
Waist-to-height	799	0.43	0.45	0.46	0.48	806	0.40	0.42	0.43	0.46
Usual energy intake [kcal/day][1]	530	1460	1615	1616	1753	812	1375	1544	1564	1742
Usual total iron intake [mg/day][1]	530	8.0	8.8	8.9	9.6	812	7.9	9.1	9.2	10.4
Usual iron intake from heme sources [mg/day][1]	530	1.2	1.4	1.4	1.8	812	1.2	1.5	1.5	1.8
Usual vitamin C intake [mg/day][1]	530	58	68	69	78	812	49	61	62	72
Boys:										
Ferritin [µg/L]	410	19.0	27	33.4	40.0	410	23.0	33	37.0	44.0
Transferrin [g/L]	410	2.60	2.8	2.84	3.10	410	2.60	2.8	2.85	3.10
Age [years]	410	4.50	6.35	6.12	7.70	410	10.30	12	11.91	13.60
BMI z-score*	410	-0.62	0.09	0.16	0.85	410	-0.37	0.42	0.45	1.27
Waist-to-height	405	0.43	0.45	0.46	0.48	406	0.40	0.43	0.44	0.46
Usual energy intake [kcal/day][1]	277	1566	1665	1688	1804	410	1443	1624	1638	1842
Usual total iron intake [mg/day][1]	277	8.5	9.1	9.2	10.0	410	8.2	9.5	9.5	10.7
Usual iron intake from heme sources [mg/day][1]	277	1.1	1.6	1.4	1.9	410	1.3	1.6	1.7	1.9
Usual vitamin C intake [mg/day][1]	277	63	73	74	83	410	48	60	61	71
Girls:										
Ferritin [µg/L]	400	24.0	31.5	35.3	43.3	402	23.0	32	35.6	44.0
Transferrin [g/L]	402	2.60	2.8	2.78	3.00	402	2.60	2.8	2.85	3.10
Age [years]	402	4.60	6.4	6.13	7.70	402	10.33	12.1	11.94	13.57
BMI z-score*	402	-0.48	0.12	0.20	0.87	402	-0.31	0.31	0.37	1.05
Waist-to-height	394	0.42	0.45	0.45	0.48	400	0.39	0.42	0.43	0.45
Usual energy intake [kcal/day][1]	253	1368	1510	1536	1681	402	1333	1473	1489	1638
Usual total iron intake [mg/day][1]	253	7.8	8.4	8.5	9.1	402	7.5	8.7	8.9	9.9
Usual iron intake from heme sources [mg/day][1]	253	1.2	1.3	1.4	1.5	402	1.0	1.3	1.4	1.7
Usual vitamin C intake [mg/day][1]	253	53	62	63	71	402	51	62	63	72

[1]Usual dietary intakes of total energy, total iron, iron from heme sources and vitamin C were estimated from 24h dietary recalls linked to food composition tables.

Abbreviations: IDEFICS: Identification and Prevention of Dietary- and Lifestyle-Induced Health Effects in Children and Infants. W0, first examination wave; W3, third examination wave.

Supplementary Table 3. Age-specific reference percentiles of serum ferritin concentrations [µg/L] in European children aged 3 to 15 years from the IDEFICS/I.Family cohort (N=3390)

Female (n=1635)						Male (n=1755)					
**Age (y)**	**P5**	**P25**	**P50**	**P75**	**P95**	**P5**	**P25**	**P50**	**P75**	**P95**	**Age (y)**
3.0	12.8	19.7	27.1	37.8	62.8	10.9	16.5	22.8	32.5	58.2	3.0
3.1	12.9	19.9	27.3	38.0	63.1	11.0	16.7	22.9	32.7	58.6	3.1
3.2	13.0	20.0	27.4	38.2	63.5	11.1	16.8	23.1	32.9	59.0	3.2
3.3	13.0	20.1	27.6	38.4	63.8	11.2	16.9	23.3	33.1	59.4	3.3
3.4	13.1	20.2	27.7	38.6	64.2	11.2	17.0	23.4	33.4	59.9	3.4
3.5	13.2	20.3	27.9	38.9	64.5	11.3	17.1	23.6	33.6	60.3	3.5
3.6	13.2	20.4	28.0	39.1	64.9	11.4	17.2	23.8	33.8	60.7	3.6
3.7	13.3	20.5	28.2	39.3	65.3	11.5	17.4	23.9	34.1	61.1	3.7
3.8	13.4	20.6	28.3	39.5	65.6	11.6	17.5	24.1	34.3	61.5	3.8
3.9	13.5	20.7	28.5	39.7	66.0	11.6	17.6	24.2	34.5	61.9	3.9
4.0	13.5	20.8	28.6	39.9	66.3	11.7	17.7	24.4	34.8	62.3	4.0
4.1	13.6	21.0	28.8	40.1	66.6	11.8	17.8	24.6	35.0	62.8	4.1
4.2	13.7	21.1	28.9	40.3	67.0	11.9	18.0	24.7	35.2	63.2	4.2
4.3	13.7	21.2	29.1	40.5	67.3	12.0	18.1	24.9	35.5	63.6	4.3
4.4	13.8	21.3	29.2	40.7	67.7	12.0	18.2	25.1	35.7	64.0	4.4
4.5	13.9	21.4	29.4	40.9	68.0	12.1	18.3	25.2	35.9	64.4	4.5
4.6	13.9	21.5	29.5	41.1	68.3	12.2	18.4	25.4	36.1	64.8	4.6
4.7	14.0	21.6	29.6	41.3	68.7	12.3	18.5	25.5	36.4	65.2	4.7
4.8	14.1	21.7	29.8	41.5	69.0	12.3	18.7	25.7	36.6	65.7	4.8
4.9	14.1	21.8	29.9	41.7	69.3	12.4	18.8	25.9	36.8	66.1	4.9
5.0	14.2	21.9	30.1	41.9	69.6	12.5	18.9	26.0	37.1	66.5	5.0
5.1	14.3	22.0	30.2	42.1	69.9	12.6	19.0	26.2	37.3	66.9	5.1
5.2	14.3	22.1	30.3	42.3	70.3	12.7	19.1	26.3	37.5	67.3	5.2
5.3	14.4	22.2	30.5	42.5	70.6	12.7	19.2	26.5	37.8	67.7	5.3

5.4	14.5	22.3	30.6	42.6	70.9	12.8	19.4	26.7	38.0	68.1	5.4
5.5	14.5	22.4	30.7	42.8	71.1	12.9	19.5	26.8	38.2	68.5	5.5
5.6	14.6	22.5	30.8	43.0	71.4	13.0	19.6	27.0	38.4	68.9	5.6
5.7	14.6	22.5	31.0	43.2	71.7	13.0	19.7	27.1	38.7	69.3	5.7
5.8	14.7	22.6	31.1	43.3	72.0	13.1	19.8	27.3	38.9	69.7	5.8
5.9	14.7	22.7	31.2	43.5	72.3	13.2	19.9	27.5	39.1	70.1	5.9
6.0	14.8	22.8	31.3	43.7	72.5	13.3	20.0	27.6	39.3	70.5	6.0
6.1	14.9	22.9	31.4	43.8	72.8	13.3	20.1	27.8	39.5	70.9	6.1
6.2	14.9	23.0	31.5	44.0	73.0	13.4	20.3	27.9	39.8	71.3	6.2
6.3	15.0	23.0	31.6	44.1	73.3	13.5	20.4	28.1	40.0	71.7	6.3
6.4	15.0	23.1	31.7	44.2	73.5	13.5	20.5	28.2	40.2	72.0	6.4
6.5	15.0	23.2	31.8	44.4	73.7	13.6	20.6	28.3	40.4	72.4	6.5
6.6	15.1	23.2	31.9	44.5	73.9	13.7	20.7	28.5	40.6	72.8	6.6
6.7	15.1	23.3	32.0	44.6	74.1	13.7	20.8	28.6	40.8	73.1	6.7
6.8	15.2	23.4	32.1	44.7	74.3	13.8	20.9	28.8	41.0	73.5	6.8
6.9	15.2	23.4	32.2	44.9	74.5	13.9	21.0	28.9	41.2	73.9	6.9
7.0	15.2	23.5	32.3	45.0	74.7	13.9	21.1	29.0	41.4	74.2	7.0
7.1	15.3	23.5	32.3	45.1	74.9	14.0	21.2	29.2	41.6	74.5	7.1
7.2	15.3	23.6	32.4	45.2	75.0	14.1	21.3	29.3	41.7	74.9	7.2
7.3	15.3	23.6	32.5	45.3	75.2	14.1	21.4	29.4	41.9	75.2	7.3
7.4	15.4	23.7	32.5	45.4	75.3	14.2	21.5	29.6	42.1	75.5	7.4
7.5	15.4	23.7	32.6	45.4	75.5	14.2	21.5	29.7	42.3	75.8	7.5
7.6	15.4	23.8	32.6	45.5	75.6	14.3	21.6	29.8	42.4	76.1	7.6
7.7	15.5	23.8	32.7	45.6	75.7	14.4	21.7	29.9	42.6	76.4	7.7
7.8	15.5	23.8	32.7	45.6	75.8	14.4	21.8	30.0	42.8	76.7	7.8
7.9	15.5	23.9	32.8	45.7	75.9	14.5	21.9	30.1	42.9	77.0	7.9
8.0	15.5	23.9	32.8	45.7	76.0	14.5	22.0	30.2	43.1	77.2	8.0
8.1	15.5	23.9	32.8	45.8	76.1	14.6	22.0	30.3	43.2	77.5	8.1
8.2	15.5	23.9	32.9	45.8	76.1	14.6	22.1	30.4	43.4	77.8	8.2

8.3	15.5	24.0	32.9	45.9	76.2	14.7	22.2	30.5	43.5	78.0	8.3
8.4	15.6	24.0	32.9	45.9	76.2	14.7	22.2	30.6	43.6	78.2	8.4
8.5	15.6	24.0	32.9	45.9	76.2	14.7	22.3	30.7	43.8	78.5	8.5
8.6	15.6	24.0	32.9	45.9	76.3	14.8	22.4	30.8	43.9	78.7	8.6
8.7	15.6	24.0	32.9	45.9	76.3	14.8	22.4	30.9	44.0	78.9	8.7
8.8	15.6	24.0	32.9	45.9	76.2	14.9	22.5	31.0	44.1	79.1	8.8
8.9	15.6	24.0	32.9	45.9	76.2	14.9	22.5	31.0	44.2	79.3	8.9
9.0	15.5	24.0	32.9	45.9	76.2	14.9	22.6	31.1	44.3	79.5	9.0
9.1	15.5	23.9	32.9	45.8	76.2	15.0	22.6	31.2	44.4	79.6	9.1
9.2	15.5	23.9	32.9	45.8	76.1	15.0	22.7	31.2	44.5	79.8	9.2
9.3	15.5	23.9	32.8	45.8	76.0	15.0	22.7	31.3	44.6	80.0	9.3
9.4	15.5	23.9	32.8	45.7	76.0	15.1	22.8	31.4	44.7	80.1	9.4
9.5	15.5	23.9	32.8	45.7	75.9	15.1	22.8	31.4	44.7	80.2	9.5
9.6	15.5	23.8	32.7	45.6	75.8	15.1	22.8	31.5	44.8	80.4	9.6
9.7	15.4	23.8	32.7	45.5	75.6	15.1	22.9	31.5	44.9	80.5	9.7
9.8	15.4	23.7	32.6	45.5	75.5	15.1	22.9	31.5	44.9	80.6	9.8
9.9	15.4	23.7	32.5	45.4	75.4	15.2	22.9	31.6	45.0	80.7	9.9
10.0	15.4	23.7	32.5	45.3	75.2	15.2	23.0	31.6	45.0	80.8	10.0
10.1	15.3	23.6	32.4	45.2	75.1	15.2	23.0	31.6	45.1	80.8	10.1
10.2	15.3	23.6	32.3	45.1	74.9	15.2	23.0	31.7	45.1	80.9	10.2
10.3	15.2	23.5	32.3	45.0	74.7	15.2	23.0	31.7	45.1	81.0	10.3
10.4	15.2	23.4	32.2	44.9	74.5	15.2	23.0	31.7	45.2	81.0	10.4
10.5	15.2	23.4	32.1	44.7	74.3	15.2	23.0	31.7	45.2	81.0	10.5
10.6	15.1	23.3	32.0	44.6	74.1	15.2	23.0	31.7	45.2	81.0	10.6
10.7	15.1	23.2	31.9	44.5	73.9	15.2	23.0	31.7	45.2	81.1	10.7
10.8	15.0	23.2	31.8	44.3	73.7	15.2	23.0	31.7	45.2	81.1	10.8
10.9	15.0	23.1	31.7	44.2	73.4	15.2	23.0	31.7	45.2	81.1	10.9
11.0	14.9	23.0	31.6	44.0	73.2	15.2	23.0	31.7	45.2	81.0	11.0
11.1	14.9	22.9	31.5	43.9	72.9	15.2	23.0	31.7	45.2	81.0	11.1

11.2	14.8	22.8	31.4	43.7	72.6	15.2	23.0	31.7	45.1	81.0	11.2
11.3	14.8	22.8	31.2	43.6	72.4	15.2	23.0	31.7	45.1	80.9	11.3
11.4	14.7	22.7	31.1	43.4	72.1	15.2	23.0	31.7	45.1	80.9	11.4
11.5	14.7	22.6	31.0	43.2	71.8	15.2	23.0	31.6	45.1	80.8	11.5
11.6	14.6	22.5	30.9	43.0	71.5	15.2	22.9	31.6	45.0	80.7	11.6
11.7	14.5	22.4	30.7	42.9	71.2	15.2	22.9	31.6	45.0	80.6	11.7
11.8	14.5	22.3	30.6	42.7	70.9	15.1	22.9	31.5	44.9	80.6	11.8
11.9	14.4	22.2	30.5	42.5	70.6	15.1	22.9	31.5	44.9	80.5	11.9
12.0	14.3	22.1	30.3	42.3	70.3	15.1	22.8	31.5	44.8	80.3	12.0
12.1	14.3	22.0	30.2	42.1	69.9	15.1	22.8	31.4	44.7	80.2	12.1
12.2	14.2	21.9	30.1	41.9	69.6	15.1	22.8	31.4	44.7	80.1	12.2
12.3	14.1	21.8	29.9	41.7	69.3	15.0	22.7	31.3	44.6	80.0	12.3
12.4	14.1	21.7	29.8	41.5	68.9	15.0	22.7	31.3	44.5	79.9	12.4
12.5	14.0	21.6	29.6	41.3	68.6	15.0	22.7	31.2	44.5	79.7	12.5
12.6	13.9	21.5	29.5	41.1	68.2	15.0	22.6	31.2	44.4	79.6	12.6
12.7	13.9	21.3	29.3	40.9	67.9	14.9	22.6	31.1	44.3	79.4	12.7
12.8	13.8	21.2	29.2	40.6	67.5	14.9	22.5	31.0	44.2	79.3	12.8
12.9	13.7	21.1	29.0	40.4	67.2	14.9	22.5	31.0	44.1	79.1	12.9
13.0	13.6	21.0	28.8	40.2	66.8	14.8	22.4	30.9	44.0	79.0	13.0
13.1	13.6	20.9	28.7	40.0	66.4	14.8	22.4	30.9	44.0	78.8	13.1
13.2	13.5	20.8	28.5	39.8	66.1	14.8	22.4	30.8	43.9	78.7	13.2
13.3	13.4	20.7	28.4	39.6	65.7	14.8	22.3	30.7	43.8	78.5	13.3
13.4	13.3	20.5	28.2	39.3	65.3	14.7	22.3	30.7	43.7	78.3	13.4
13.5	13.3	20.4	28.1	39.1	65.0	14.7	22.2	30.6	43.6	78.2	13.5
13.6	13.2	20.3	27.9	38.9	64.6	14.7	22.2	30.5	43.5	78.0	13.6
13.7	13.1	20.2	27.7	38.7	64.2	14.6	22.1	30.5	43.4	77.8	13.7
13.8	13.0	20.1	27.6	38.4	63.9	14.6	22.1	30.4	43.3	77.7	13.8
13.9	13.0	20.0	27.4	38.2	63.5	14.6	22.0	30.3	43.2	77.5	13.9
14.0	12.9	19.9	27.3	38.0	63.1	14.5	22.0	30.3	43.1	77.4	14.0

14.1	12.8	19.7	27.1	37.8	62.8	14.5	21.9	30.2	43.0	77.2	14.1
14.2	12.7	19.6	26.9	37.6	62.4	14.5	21.9	30.2	42.9	77.0	14.2
14.3	12.7	19.5	26.8	37.3	62.0	14.4	21.8	30.1	42.9	76.9	14.3
14.4	12.6	19.4	26.6	37.1	61.6	14.4	21.8	30.0	42.8	76.7	14.4
14.5	12.5	19.3	26.5	36.9	61.3	14.4	21.7	30.0	42.7	76.5	14.5
14.6	12.4	19.2	26.3	36.7	60.9	14.4	21.7	29.9	42.6	76.4	14.6
14.7	12.4	19.0	26.1	36.4	60.5	14.3	21.7	29.8	42.5	76.2	14.7
14.8	12.3	18.9	26.0	36.2	60.2	14.3	21.6	29.8	42.4	76.0	14.8
14.9	12.2	18.8	25.8	36.0	59.8	14.3	21.6	29.7	42.3	75.9	14.9
15.0	12.1	18.7	25.7	35.8	59.4	14.2	21.5	29.6	42.2	75.7	15.0

Abbreviation: IDEFICS: Identification and Prevention of Dietary- and Lifestyle-Induced Health Effects in Children and Infants.

Supplementary Table 4. BMI-specific reference percentiles of serum ferritin concentrations [µg/L] in European children aged 3 to 15 years from the IDEFICS/I.Family cohort (N=3390)

Female (n=1635)						Male (n=1755)					
**BMI z-score**	**P5**	**P25**	**P50**	**P75**	**P95**	**P5**	**P25**	**P50**	**P75**	**P95**	**BMI z-score**
-2.0	13.6	21.3	29.5	41.4	68.5	14.1	21.6	30.0	42.9	76.1	-2.0
-1.9	13.6	21.4	29.6	41.4	68.6	14.1	21.6	30.0	42.9	76.1	-1.9
-1.8	13.6	21.4	29.6	41.5	68.7	14.1	21.6	30.0	42.8	76.1	-1.8
-1.7	13.6	21.4	29.7	41.6	68.8	14.1	21.6	30.0	42.8	76.0	-1.7
-1.6	13.7	21.5	29.7	41.6	68.9	14.0	21.6	29.9	42.8	75.9	-1.6
-1.5	13.7	21.5	29.8	41.7	69.0	14.0	21.5	29.9	42.7	75.8	-1.5
-1.4	13.7	21.5	29.8	41.7	69.1	14.0	21.5	29.8	42.6	75.6	-1.4
-1.3	13.7	21.6	29.9	41.8	69.2	14.0	21.4	29.8	42.5	75.4	-1.3
-1.2	13.7	21.6	29.9	41.9	69.3	13.9	21.4	29.7	42.4	75.2	-1.2
-1.1	13.8	21.6	29.9	41.9	69.4	13.9	21.3	29.6	42.2	74.9	-1.1
-1.0	13.8	21.6	30.0	42.0	69.5	13.8	21.2	29.4	42.0	74.6	-1.0
-0.9	13.8	21.7	30.0	42.0	69.6	13.8	21.1	29.3	41.9	74.3	-0.9
-0.8	13.8	21.7	30.1	42.1	69.7	13.7	21.0	29.2	41.7	74.0	-0.8
-0.7	13.8	21.7	30.1	42.2	69.8	13.6	20.9	29.1	41.5	73.7	-0.7
-0.6	13.9	21.8	30.2	42.2	69.9	13.6	20.9	28.9	41.3	73.4	-0.6
-0.5	13.9	21.8	30.2	42.3	70.0	13.5	20.8	28.8	41.2	73.1	-0.5
-0.4	13.9	21.8	30.2	42.3	70.1	13.5	20.7	28.7	41.0	72.8	-0.4
-0.3	13.9	21.9	30.3	42.4	70.2	13.4	20.6	28.6	40.9	72.6	-0.3
-0.2	13.9	21.9	30.3	42.5	70.3	13.4	20.6	28.5	40.7	72.3	-0.2
-0.1	14.0	21.9	30.4	42.5	70.4	13.4	20.5	28.5	40.6	72.2	-0.1
0.0	14.0	22.0	30.4	42.6	70.5	13.3	20.5	28.4	40.6	72.0	0.0
0.1	14.0	22.0	30.5	42.6	70.6	13.3	20.5	28.4	40.5	72.0	0.1
0.2	14.0	22.0	30.5	42.7	70.7	13.3	20.5	28.4	40.5	72.0	0.2
0.3	14.0	22.1	30.5	42.8	70.8	13.3	20.5	28.4	40.6	72.0	0.3

0.4	14.1	22.1	30.6	42.8	70.9	13.4	20.5	28.5	40.6	72.2	0.4
0.5	14.1	22.1	30.6	42.9	71.0	13.4	20.6	28.5	40.7	72.3	0.5
0.6	14.1	22.1	30.7	42.9	71.1	13.4	20.6	28.6	40.9	72.6	0.6
0.7	14.1	22.2	30.7	43.0	71.2	13.5	20.7	28.8	41.1	72.9	0.7
0.8	14.1	22.2	30.8	43.1	71.3	13.6	20.8	28.9	41.3	73.3	0.8
0.9	14.2	22.2	30.8	43.1	71.4	13.7	21.0	29.1	41.5	73.8	0.9
1.0	14.2	22.3	30.8	43.2	71.5	13.7	21.1	29.3	41.8	74.3	1.0
1.1	14.2	22.3	30.9	43.3	71.6	13.9	21.3	29.5	42.2	74.9	1.1
1.2	14.2	22.3	30.9	43.3	71.7	14.0	21.5	29.8	42.6	75.5	1.2
1.3	14.2	22.4	31.0	43.4	71.8	14.1	21.7	30.1	43.0	76.3	1.3
1.4	14.3	22.4	31.0	43.4	71.9	14.3	21.9	30.4	43.5	77.2	1.4
1.5	14.3	22.4	31.1	43.5	72.0	14.5	22.2	30.8	44.0	78.1	1.5
1.6	14.3	22.5	31.1	43.6	72.1	14.7	22.5	31.3	44.6	79.2	1.6
1.7	14.3	22.5	31.2	43.6	72.2	14.9	22.9	31.7	45.3	80.4	1.7
1.8	14.3	22.5	31.2	43.7	72.3	15.1	23.2	32.2	46.0	81.7	1.8
1.9	14.4	22.6	31.2	43.7	72.4	15.4	23.6	32.7	46.7	83.0	1.9
2.0	14.4	22.6	31.3	43.8	72.5	15.6	24.0	33.3	47.5	84.4	2.0
2.1	14.4	22.6	31.3	43.9	72.6	15.9	24.4	33.9	48.4	85.8	2.1
2.2	14.4	22.6	31.4	43.9	72.7	16.2	24.8	34.5	49.2	87.4	2.2
2.3	14.4	22.7	31.4	44.0	72.8	16.5	25.3	35.1	50.1	88.9	2.3
2.4	14.5	22.7	31.5	44.0	72.9	16.8	25.7	35.7	51.0	90.5	2.4
2.5	14.5	22.7	31.5	44.1	73.0	17.1	26.2	36.4	51.9	92.2	2.5
2.6	14.5	22.8	31.5	44.2	73.1	17.4	26.7	37.0	52.9	93.8	2.6
2.7	14.5	22.8	31.6	44.2	73.2	17.7	27.2	37.7	53.8	95.5	2.7
2.8	14.5	22.8	31.6	44.3	73.3	18.0	27.6	38.4	54.8	97.2	2.8
2.9	14.6	22.9	31.7	44.3	73.4	18.3	28.1	39.0	55.7	98.9	2.9
3.0	14.6	22.9	31.7	44.4	73.5	18.6	28.6	39.7	56.7	100.6	3.0

Abbreviation: IDEFICS: Identification and Prevention of Dietary- and Lifestyle-Induced Health Effects in Children and Infants.

Supplementary Table 5. Age-specific reference percentiles of serum transferrin concentrations [g/L] in European children aged 3 to 15 years from the IDEFICS/I.Family cohort (N=3416)

Female (n=1656)						Male (n=1760)					
Age (y)	P5	P25	P50	P75	P95	P5	P25	P50	P75	P95	Age (y)
3.0	2.24	2.57	2.78	3.00	3.39	2.31	2.61	2.84	3.08	3.46	3.0
3.1	2.24	2.57	2.78	3.00	3.39	2.31	2.61	2.84	3.08	3.46	3.1
3.2	2.24	2.57	2.78	3.00	3.39	2.31	2.61	2.84	3.08	3.46	3.2
3.3	2.24	2.56	2.78	3.00	3.39	2.31	2.61	2.84	3.08	3.45	3.3
3.4	2.24	2.56	2.77	3.00	3.39	2.31	2.61	2.84	3.08	3.45	3.4
3.5	2.24	2.56	2.77	3.00	3.38	2.31	2.61	2.83	3.08	3.45	3.5
3.6	2.24	2.56	2.77	3.00	3.38	2.31	2.61	2.83	3.07	3.45	3.6
3.7	2.24	2.56	2.77	2.99	3.38	2.31	2.61	2.83	3.07	3.45	3.7
3.8	2.24	2.56	2.77	2.99	3.38	2.31	2.61	2.83	3.07	3.45	3.8
3.9	2.24	2.56	2.77	2.99	3.38	2.31	2.61	2.83	3.07	3.45	3.9
4.0	2.24	2.56	2.77	2.99	3.38	2.31	2.60	2.83	3.07	3.45	4.0
4.1	2.24	2.56	2.77	2.99	3.38	2.31	2.60	2.83	3.07	3.45	4.1
4.2	2.24	2.56	2.77	2.99	3.38	2.30	2.60	2.83	3.07	3.44	4.2
4.3	2.24	2.56	2.77	2.99	3.38	2.30	2.60	2.83	3.07	3.44	4.3
4.4	2.24	2.56	2.77	2.99	3.38	2.30	2.60	2.83	3.07	3.44	4.4
4.5	2.24	2.56	2.77	2.99	3.38	2.30	2.60	2.82	3.07	3.44	4.5
4.6	2.24	2.56	2.77	2.99	3.38	2.30	2.60	2.82	3.06	3.44	4.6
4.7	2.24	2.56	2.77	2.99	3.38	2.30	2.60	2.82	3.06	3.44	4.7
4.8	2.24	2.56	2.77	2.99	3.38	2.30	2.60	2.82	3.06	3.44	4.8
4.9	2.24	2.56	2.77	2.99	3.38	2.30	2.60	2.82	3.06	3.44	4.9
5.0	2.24	2.56	2.77	2.99	3.38	2.30	2.60	2.82	3.06	3.43	5.0
5.1	2.24	2.56	2.77	2.99	3.38	2.30	2.59	2.82	3.06	3.43	5.1
5.2	2.24	2.56	2.77	2.99	3.38	2.30	2.59	2.82	3.06	3.43	5.2
5.3	2.23	2.56	2.77	2.99	3.37	2.30	2.59	2.82	3.06	3.43	5.3

5.4	2.23	2.56	2.77	2.99	3.37	2.30	2.59	2.82	3.06	3.43	5.4
5.5	2.23	2.56	2.76	2.99	3.37	2.29	2.59	2.82	3.06	3.43	5.5
5.6	2.23	2.55	2.76	2.99	3.37	2.29	2.59	2.81	3.05	3.43	5.6
5.7	2.23	2.55	2.76	2.99	3.37	2.29	2.59	2.81	3.05	3.43	5.7
5.8	2.23	2.55	2.76	2.98	3.37	2.29	2.59	2.81	3.05	3.43	5.8
5.9	2.23	2.55	2.76	2.98	3.37	2.29	2.59	2.81	3.05	3.43	5.9
6.0	2.23	2.55	2.76	2.98	3.37	2.29	2.59	2.81	3.05	3.42	6.0
6.1	2.23	2.55	2.76	2.98	3.37	2.29	2.59	2.81	3.05	3.42	6.1
6.2	2.23	2.55	2.76	2.98	3.37	2.29	2.59	2.81	3.05	3.42	6.2
6.3	2.23	2.55	2.76	2.98	3.37	2.29	2.59	2.81	3.05	3.42	6.3
6.4	2.23	2.55	2.76	2.98	3.37	2.29	2.58	2.81	3.05	3.42	6.4
6.5	2.23	2.55	2.76	2.98	3.37	2.29	2.58	2.81	3.05	3.42	6.5
6.6	2.23	2.55	2.76	2.98	3.37	2.29	2.58	2.81	3.05	3.42	6.6
6.7	2.23	2.55	2.76	2.98	3.37	2.29	2.58	2.81	3.04	3.42	6.7
6.8	2.23	2.55	2.76	2.98	3.37	2.29	2.58	2.80	3.04	3.42	6.8
6.9	2.23	2.55	2.76	2.98	3.37	2.29	2.58	2.80	3.04	3.42	6.9
7.0	2.23	2.55	2.76	2.98	3.37	2.29	2.58	2.80	3.04	3.41	7.0
7.1	2.23	2.55	2.76	2.99	3.37	2.28	2.58	2.80	3.04	3.41	7.1
7.2	2.23	2.55	2.76	2.99	3.37	2.28	2.58	2.80	3.04	3.41	7.2
7.3	2.23	2.56	2.76	2.99	3.37	2.28	2.58	2.80	3.04	3.41	7.3
7.4	2.23	2.56	2.77	2.99	3.37	2.28	2.58	2.80	3.04	3.41	7.4
7.5	2.24	2.56	2.77	2.99	3.37	2.28	2.58	2.80	3.04	3.41	7.5
7.6	2.24	2.56	2.77	2.99	3.38	2.28	2.58	2.80	3.04	3.41	7.6
7.7	2.24	2.56	2.77	2.99	3.38	2.28	2.58	2.80	3.04	3.41	7.7
7.8	2.24	2.56	2.77	2.99	3.38	2.28	2.58	2.80	3.04	3.41	7.8
7.9	2.24	2.56	2.77	2.99	3.38	2.28	2.58	2.80	3.04	3.41	7.9
8.0	2.24	2.56	2.77	2.99	3.38	2.28	2.58	2.80	3.04	3.41	8.0
8.1	2.24	2.56	2.77	2.99	3.38	2.28	2.57	2.80	3.04	3.41	8.1
8.2	2.24	2.56	2.77	2.99	3.38	2.28	2.57	2.80	3.04	3.41	8.2

8.3	2.24	2.56	2.77	2.99	3.38	2.28	2.57	2.80	3.03	3.41	8.3
8.4	2.24	2.56	2.77	2.99	3.38	2.28	2.57	2.80	3.03	3.41	8.4
8.5	2.24	2.56	2.77	2.99	3.38	2.28	2.57	2.80	3.03	3.41	8.5
8.6	2.24	2.56	2.77	3.00	3.39	2.28	2.57	2.80	3.03	3.41	8.6
8.7	2.24	2.57	2.78	3.00	3.39	2.28	2.57	2.80	3.03	3.41	8.7
8.8	2.24	2.57	2.78	3.00	3.39	2.28	2.57	2.80	3.03	3.41	8.8
8.9	2.24	2.57	2.78	3.00	3.39	2.28	2.57	2.80	3.03	3.41	8.9
9.0	2.25	2.57	2.78	3.00	3.39	2.28	2.57	2.80	3.03	3.41	9.0
9.1	2.25	2.57	2.78	3.00	3.39	2.28	2.57	2.80	3.03	3.41	9.1
9.2	2.25	2.57	2.78	3.00	3.39	2.28	2.57	2.80	3.03	3.41	9.2
9.3	2.25	2.57	2.78	3.01	3.40	2.28	2.57	2.80	3.03	3.41	9.3
9.4	2.25	2.57	2.79	3.01	3.40	2.28	2.57	2.80	3.03	3.41	9.4
9.5	2.25	2.58	2.79	3.01	3.40	2.28	2.57	2.80	3.03	3.41	9.5
9.6	2.25	2.58	2.79	3.01	3.40	2.28	2.57	2.80	3.03	3.41	9.6
9.7	2.25	2.58	2.79	3.01	3.40	2.28	2.57	2.80	3.04	3.41	9.7
9.8	2.26	2.58	2.79	3.01	3.41	2.28	2.58	2.80	3.04	3.41	9.8
9.9	2.26	2.58	2.79	3.02	3.41	2.28	2.58	2.80	3.04	3.41	9.9
10.0	2.26	2.58	2.80	3.02	3.41	2.28	2.58	2.80	3.04	3.41	10.0
10.1	2.26	2.58	2.80	3.02	3.41	2.28	2.58	2.80	3.04	3.41	10.1
10.2	2.26	2.59	2.80	3.02	3.42	2.28	2.58	2.80	3.04	3.41	10.2
10.3	2.26	2.59	2.80	3.02	3.42	2.28	2.58	2.80	3.04	3.41	10.3
10.4	2.26	2.59	2.80	3.03	3.42	2.28	2.58	2.80	3.04	3.41	10.4
10.5	2.27	2.59	2.80	3.03	3.42	2.29	2.58	2.80	3.04	3.42	10.5
10.6	2.27	2.59	2.81	3.03	3.42	2.29	2.58	2.81	3.04	3.42	10.6
10.7	2.27	2.60	2.81	3.03	3.43	2.29	2.58	2.81	3.05	3.42	10.7
10.8	2.27	2.60	2.81	3.04	3.43	2.29	2.58	2.81	3.05	3.42	10.8
10.9	2.27	2.60	2.81	3.04	3.43	2.29	2.59	2.81	3.05	3.42	10.9
11.0	2.27	2.60	2.81	3.04	3.43	2.29	2.59	2.81	3.05	3.42	11.0
11.1	2.28	2.60	2.82	3.04	3.44	2.29	2.59	2.81	3.05	3.43	11.1

11.2	2.28	2.61	2.82	3.04	3.44	2.29	2.59	2.81	3.05	3.43	11.2
11.3	2.28	2.61	2.82	3.05	3.44	2.30	2.59	2.82	3.06	3.43	11.3
11.4	2.28	2.61	2.82	3.05	3.44	2.30	2.59	2.82	3.06	3.43	11.4
11.5	2.28	2.61	2.83	3.05	3.45	2.30	2.60	2.82	3.06	3.44	11.5
11.6	2.28	2.61	2.83	3.05	3.45	2.30	2.60	2.82	3.06	3.44	11.6
11.7	2.29	2.61	2.83	3.06	3.45	2.30	2.60	2.83	3.07	3.44	11.7
11.8	2.29	2.62	2.83	3.06	3.46	2.31	2.60	2.83	3.07	3.44	11.8
11.9	2.29	2.62	2.83	3.06	3.46	2.31	2.61	2.83	3.07	3.45	11.9
12.0	2.29	2.62	2.84	3.06	3.46	2.31	2.61	2.83	3.07	3.45	12.0
12.1	2.29	2.62	2.84	3.06	3.46	2.31	2.61	2.84	3.08	3.45	12.1
12.2	2.30	2.62	2.84	3.07	3.47	2.31	2.61	2.84	3.08	3.46	12.2
12.3	2.30	2.63	2.84	3.07	3.47	2.32	2.61	2.84	3.08	3.46	12.3
12.4	2.30	2.63	2.84	3.07	3.47	2.32	2.62	2.84	3.09	3.46	12.4
12.5	2.30	2.63	2.85	3.07	3.47	2.32	2.62	2.85	3.09	3.47	12.5
12.6	2.30	2.63	2.85	3.08	3.48	2.32	2.62	2.85	3.09	3.47	12.6
12.7	2.30	2.63	2.85	3.08	3.48	2.32	2.63	2.85	3.10	3.47	12.7
12.8	2.31	2.64	2.85	3.08	3.48	2.33	2.63	2.86	3.10	3.48	12.8
12.9	2.31	2.64	2.86	3.08	3.48	2.33	2.63	2.86	3.10	3.48	12.9
13.0	2.31	2.64	2.86	3.09	3.49	2.33	2.63	2.86	3.10	3.49	13.0
13.1	2.31	2.64	2.86	3.09	3.49	2.33	2.64	2.86	3.11	3.49	13.1
13.2	2.31	2.64	2.86	3.09	3.49	2.34	2.64	2.87	3.11	3.49	13.2
13.3	2.31	2.65	2.86	3.09	3.49	2.34	2.64	2.87	3.11	3.50	13.3
13.4	2.32	2.65	2.87	3.09	3.50	2.34	2.64	2.87	3.12	3.50	13.4
13.5	2.32	2.65	2.87	3.10	3.50	2.34	2.65	2.88	3.12	3.50	13.5
13.6	2.32	2.65	2.87	3.10	3.50	2.35	2.65	2.88	3.13	3.51	13.6
13.7	2.32	2.65	2.87	3.10	3.50	2.35	2.65	2.88	3.13	3.51	13.7
13.8	2.32	2.66	2.87	3.10	3.51	2.35	2.66	2.89	3.13	3.52	13.8
13.9	2.32	2.66	2.88	3.11	3.51	2.36	2.66	2.89	3.14	3.52	13.9
14.0	2.33	2.66	2.88	3.11	3.51	2.36	2.66	2.89	3.14	3.52	14.0

14.1	2.33	2.66	2.88	3.11	3.51	2.36	2.67	2.90	3.14	3.53	14.1
14.2	2.33	2.66	2.88	3.11	3.52	2.36	2.67	2.90	3.15	3.53	14.2
14.3	2.33	2.66	2.88	3.11	3.52	2.37	2.67	2.90	3.15	3.53	14.3
14.4	2.33	2.67	2.89	3.12	3.52	2.37	2.67	2.90	3.15	3.54	14.4
14.5	2.33	2.67	2.89	3.12	3.52	2.37	2.68	2.91	3.16	3.54	14.5
14.6	2.33	2.67	2.89	3.12	3.53	2.37	2.68	2.91	3.16	3.55	14.6
14.7	2.34	2.67	2.89	3.12	3.53	2.38	2.68	2.91	3.16	3.55	14.7
14.8	2.34	2.67	2.89	3.12	3.53	2.38	2.69	2.92	3.17	3.55	14.8
14.9	2.34	2.68	2.89	3.13	3.53	2.38	2.69	2.92	3.17	3.56	14.9
15.0	2.34	2.68	2.90	3.13	3.53	2.38	2.69	2.92	3.17	3.56	15.0

Abbreviation: IDEFICS: Identification and Prevention of Dietary- and Lifestyle-Induced Health Effects in Children and Infants.

Supplementary Table 6. BMI-specific reference percentiles of serum transferrin concentrations [g/L] in European children aged 3 to 15 years from the IDEFICS/I.Family cohort (N=3416)

Female (n=1656)						Male (n=1760)					
BMI z-score	P5	P25	P50	P75	P95	P5	P25	P50	P75	P95	BMI z-score
-2.0	2.16	2.46	2.66	2.88	3.25	2.28	2.54	2.72	2.92	3.23	-2.0
-1.9	2.17	2.47	2.67	2.88	3.26	2.28	2.54	2.73	2.93	3.24	-1.9
-1.8	2.17	2.48	2.68	2.89	3.26	2.28	2.54	2.73	2.93	3.24	-1.8
-1.7	2.17	2.48	2.68	2.90	3.27	2.29	2.54	2.74	2.94	3.25	-1.7
-1.6	2.18	2.49	2.69	2.90	3.28	2.29	2.55	2.74	2.95	3.26	-1.6
-1.5	2.18	2.49	2.70	2.91	3.29	2.29	2.55	2.75	2.95	3.27	-1.5
-1.4	2.19	2.50	2.70	2.92	3.29	2.29	2.55	2.75	2.96	3.28	-1.4
-1.3	2.19	2.50	2.71	2.92	3.30	2.29	2.56	2.76	2.97	3.29	-1.3
-1.2	2.20	2.51	2.71	2.93	3.31	2.29	2.56	2.76	2.97	3.30	-1.2
-1.1	2.20	2.51	2.72	2.94	3.32	2.29	2.56	2.76	2.98	3.31	-1.1
-1.0	2.21	2.52	2.73	2.94	3.32	2.29	2.57	2.77	2.98	3.31	-1.0
-0.9	2.21	2.53	2.73	2.95	3.33	2.30	2.57	2.77	2.99	3.32	-0.9
-0.8	2.22	2.53	2.74	2.96	3.34	2.30	2.57	2.78	3.00	3.33	-0.8
-0.7	2.22	2.54	2.74	2.96	3.35	2.30	2.58	2.78	3.00	3.34	-0.7
-0.6	2.23	2.54	2.75	2.97	3.35	2.30	2.58	2.79	3.01	3.35	-0.6
-0.5	2.23	2.55	2.76	2.98	3.36	2.30	2.58	2.79	3.02	3.36	-0.5
-0.4	2.24	2.55	2.76	2.98	3.37	2.30	2.59	2.80	3.02	3.37	-0.4
-0.3	2.24	2.56	2.77	2.99	3.38	2.30	2.59	2.80	3.03	3.38	-0.3
-0.2	2.25	2.57	2.77	3.00	3.38	2.30	2.59	2.81	3.03	3.39	-0.2
-0.1	2.25	2.57	2.78	3.00	3.39	2.30	2.59	2.81	3.04	3.40	-0.1
0.0	2.26	2.58	2.79	3.01	3.40	2.31	2.60	2.82	3.05	3.41	0.0
0.1	2.26	2.58	2.79	3.01	3.41	2.31	2.60	2.82	3.05	3.41	0.1
0.2	2.27	2.59	2.80	3.02	3.41	2.31	2.60	2.82	3.06	3.42	0.2
0.3	2.27	2.59	2.80	3.03	3.42	2.31	2.61	2.83	3.07	3.43	0.3

0.4	2.28	2.60	2.81	3.03	3.43	2.31	2.61	2.83	3.07	3.44	0.4
0.5	2.28	2.60	2.82	3.04	3.43	2.31	2.61	2.84	3.08	3.45	0.5
0.6	2.29	2.61	2.82	3.05	3.44	2.31	2.62	2.84	3.09	3.46	0.6
0.7	2.29	2.62	2.83	3.05	3.45	2.31	2.62	2.85	3.09	3.47	0.7
0.8	2.30	2.62	2.83	3.06	3.46	2.31	2.62	2.85	3.10	3.48	0.8
0.9	2.30	2.63	2.84	3.07	3.46	2.31	2.62	2.86	3.11	3.49	0.9
1.0	2.31	2.63	2.85	3.07	3.47	2.31	2.63	2.86	3.11	3.50	1.0
1.1	2.31	2.64	2.85	3.08	3.48	2.32	2.63	2.87	3.12	3.51	1.1
1.2	2.32	2.64	2.86	3.09	3.49	2.32	2.63	2.87	3.13	3.52	1.2
1.3	2.32	2.65	2.87	3.09	3.49	2.32	2.64	2.88	3.13	3.53	1.3
1.4	2.33	2.66	2.87	3.10	3.50	2.32	2.64	2.88	3.14	3.54	1.4
1.5	2.33	2.66	2.88	3.11	3.51	2.32	2.64	2.88	3.14	3.55	1.5
1.6	2.34	2.67	2.88	3.11	3.52	2.32	2.64	2.89	3.15	3.56	1.6
1.7	2.34	2.67	2.89	3.12	3.52	2.32	2.65	2.89	3.16	3.57	1.7
1.8	2.35	2.68	2.90	3.13	3.53	2.32	2.65	2.90	3.16	3.58	1.8
1.9	2.35	2.68	2.90	3.13	3.54	2.32	2.65	2.90	3.17	3.59	1.9
2.0	2.36	2.69	2.91	3.14	3.55	2.32	2.66	2.91	3.18	3.60	2.0
2.1	2.36	2.69	2.91	3.15	3.55	2.32	2.66	2.91	3.18	3.61	2.1
2.2	2.37	2.70	2.92	3.15	3.56	2.32	2.66	2.92	3.19	3.62	2.2
2.3	2.37	2.71	2.93	3.16	3.57	2.32	2.66	2.92	3.20	3.63	2.3
2.4	2.38	2.71	2.93	3.17	3.58	2.32	2.67	2.93	3.20	3.64	2.4
2.5	2.38	2.72	2.94	3.17	3.58	2.32	2.67	2.93	3.21	3.65	2.5
2.6	2.39	2.72	2.94	3.18	3.59	2.32	2.67	2.94	3.22	3.66	2.6
2.7	2.39	2.73	2.95	3.19	3.60	2.32	2.67	2.94	3.23	3.67	2.7
2.8	2.40	2.73	2.96	3.19	3.61	2.32	2.68	2.94	3.23	3.68	2.8
2.9	2.40	2.74	2.96	3.20	3.61	2.32	2.68	2.95	3.24	3.69	2.9
3.0	2.41	2.74	2.97	3.20	3.62	2.32	2.68	2.95	3.25	3.70	3.0

Abbreviation: IDEFICS: Identification and Prevention of Dietary- and Lifestyle-Induced Health Effects in Children and Infants.

Supplementary Table 7: Cross-sectional associations of usual total iron intake with serum ferritin [µg/L] in girls and boys during two examination waves and sensitivity analyses in the IDEFICS/I.Family study

**Model**	**Main model**			**Sensitivity analysis including usual vitamin C intake[2]**			**Sensitivity analysis with usual iron intake from heme sources[2] as exposure**		
**Wave 0 (2007/2008)**									
Logistic Regression **(Girls: n=428, Boys: n=455)**									
**Odds ratio for having a sufficient iron status (ref.: deficient status)[1]**	OR	95%-CI		OR	95%-CI		OR	95%-CI	
**Total iron intake [mg/d][2]**									
**Girls**	1.08	0.33	4.17	1.03	0.29	4.47	1.25	0.06	35.24
**Boys**	1.48	0.95	2.40	1.47	0.91	2.48	1.28	0.76	2.14
**Mainly vegetarian diet[3]**									
**Girls**	NA	NA	NA	NA	NA	NA	NA	NA	NA
**Boys**	0.32	0.04	6.53	0.32	0.04	6.53	0.33	0.05	6.72
**Vitamin C intake [mg/d][2]**									
**Girls**				1.01	0.95	1.07			
**Boys**				1.00	0.98	1.03			
Linear Model (**Girls: n =428, Boys: n =455)**									
**β estimate**	β	95%-CI		β	95%-CI		β	95%-CI	
**Total iron intake [mg/d][2]**									
**Girls**	2.03	-0.53	4.59	2.83	0.04	5.62	9.68	3.10	16.26
**Boys**	0.73	-1.75	3.21	0.06	-2.64	2.76	2.77	-0.37	5.91
**Mainly vegetarian diet[3]**									
**Girls**	-2.38	-15.32	10.57	-2.53	-15.46	10.40	3.92	-9.70	17.54
**Boys**	-6.12	-24.67	12.44	-5.75	-24.30	-12.81	-5.09	-23.63	13.44
**Vitamin C intake [mg/d][2]**									
**Girls**				-0.09	-0.21	0.03			
**Boys**				0.09	-0.05	0.23			
**Wave 3 (2013/2014) (Girls: n =679, Boys: n =715)**									
Logistic Regression									
**Odds ratio for having a sufficient iron status (ref.: deficient status)[1]**	OR	95%-CI		OR	95%-CI		OR	95%-CI	
**Total iron intake [mg/d][2]**									
**Girls**	1.04	0.83	1.31	1.07	0.85	1.38	1.77	0.93	3.57
**Boys**	1.17	0.89	1.56	1.11	0.83	1.50	2.17	0.86	5.85
**Mainly vegetarian diet[3]**									
**Girls**	0.47	0.20	1.23	0.46	0.20	1.20	0.71	0.27	2.04
**Boys**	0.33	0.12	1.02	0.33	0.12	1.02	0.57	0.17	2.09
**Vitamin C intake [mg/d][2]**									
**Girls**				0.99	0.97	1.01			
**Boys**				1.01	0.99	1.04			
Linear Model									
**β estimate**	β	95%-CI		β	95%-CI		β	95%-CI	
**Total iron intake [mg/d][2]**									
**Girls**	-0.15	-1.25	0.95	-0.55	-1.71	0.61	2.85	0.03	5.66
**Boys**	1.11	-0.26	2.49	0.90	-0.55	2.35	5.47	1.21	9.72
**Mainly vegetarian diet[3]**									
**Girls**	-4.09	-9.50	1.31	-4.03	-9.42	1.36	-2.09	-7.83	3.66
**Boys**	-2.06	-9.16	5.04	-2.00	-9.10	5.10	2.01	-5.82	9.84
**Vitamin C intake [mg/d][2]**									
**Girls**				0.10	0.00	0.19			
**Boys**				0.05	-0.06	0.16			

Adjusted for usual energy intake, age, BMI-z-score, sex, country and ISCED

[1]Iron deficiency defined according to the WHO: children aged <5 years: serum ferritin <12 µg/L; children of 5 years or older: serum ferritin <15 µg/L.

[2]Usual dietary intakes of total iron, iron from heme sources and vitamin C were estimated from 24h dietary recalls linked to food composition tables.

[3]Children were classified as mainly vegetarian if they replied to the specific question to typically exclude meat, poultry, sausage and fish from their diet in the Food Frequency Questionnaire. Some of them however reported later on to occasionally consume meat or fish.

Abbreviations: β, β estimate; IDEFICS: Identification and Prevention of Dietary- and Lifestyle-Induced Health Effects in Children and Infants; NA, not available – number of mainly vegetarians too low; OR, Odds ratio.

Supplementary Table 8: Longitudinal associations of usual total iron intake with serum ferritin [µg/L] at W3 (2013/2014) adjusted for serum ferritin at W0 (2007/08) in girls and boys and sensitivity analyses in the IDEFICS/I.Family study

**Model**	**Main model**			**Sensitivity analysis including usual vitamin C intake**[2]			**Sensitivity analysis with usual iron intake from heme sources**[2] **as exposure**		
**Longitudinal associations (Girls: n =400, Boys: n =410)**									
Logistic Regression									
**Odds ratio for having a sufficient iron status (ref.: deficient status)**[1]	OR	95%-CI		OR	95%-CI		OR	95%-CI	
**Total iron intake [mg/d]**[2]									
**Girls**	1.19	0.89	1.66	1.29	0.93	1.87	1.75	0.74	4.74
**Boys**	0.94	0.67	1.37	0.89	0.61	1.32	2.85	0.76	12.53
**Mainly vegetarian diet**[3]									
**Girls**	0.37	0.12	1.44	0.38	0.12	1.47	0.60	0.16	2.69
**Boys**	0.29	0.08	1.39	0.30	0.08	1.42	0.60	0.13	3.48
**Vitamin C intake [mg/d]**[2]									
Girls	0			0.98	0.96	1.01	0		
Boys	0			1.02	0.98	1.05	0		
Linear Model									
**β estimate**	β	95%-CI		β	95%-CI		β	95%-CI	
**Total iron intake [mg/d]**[2]									
**Girls**	-0.46	-1.66	0.74	-0.47	-1.77	0.83	3.29	0	6.58
**Boys**	0.57	-0.96	2.11	0.57	-1.06	2.21	3.45	-1.32	8.22
**Mainly vegetarian diet**[3]									
**Girls**	-4.76	-11.5	1.98	-4.76	-11.51	1.99	-2.41	-9.57	4.75
**Boys**	-3.37	-11.63	4.9	-3.36	-11.65	4.92	-0.77	-9.83	8.29
**Vitamin C intake [mg/d]**[2]									
**Girls**	0			0	-0.12	0.12	0		
**Boys**	0			0	-0.13	0.13	0		

Models adjusted for usual energy intake, age, BMI-z-score, sex, country, ISCED (all at W3) and ferritin at W0

[1]Iron deficiency defined according to the WHO: children aged <5 years: serum ferritin <12 µg/L; children of 5 years or older: serum ferritin <15 µg/L.

[2]Usual dietary intakes of total iron, iron from heme sources and vitamin C were estimated from 24h dietary recalls linked to food composition tables

[3]Children were classified as mainly vegetarian if they replied to the specific question to typically exclude meat, poultry, sausage and fish from their diet in the Food Frequency Questionnaire. Some of them however reported later on to occasionally consume meat or fish.

Abbreviations: β, β estimate; IDEFICS: Identification and Prevention of Dietary- and Lifestyle-Induced Health Effects in Children and Infants; OR, Odds ratio; W0, first examination wave; W3, third examination wave.

Supplementary Table 9: Cross-sectional and longitudinal associations (linear model) from W0 (2007/2008) to W3 (2013/2014) of usual total iron intake with serum transferrin [g/L] in girls and boys and sensitivity analyses in the IDEFICS/I.Family study

Model	Main model			Sensitivity analysis including usual vitamin C intake[1]			Sensitivity analysis with usual iron intake from heme sources[1] as exposure		
**Wave 0 (Girls n =428, Boys n =455)**									
**β estimate**	β	95%-CI		β	95%-CI		β	95%-CI	
**Total iron intake [mg/d][1]**									
**Girls**	0.01	-0.05	0.07	0.02	-0.04	0.08	0.08	-0.07	0.23
**Boys**	-0.01	-0.05	0.03	0	-0.04	0.05	-0.04	-0.09	0.01
**Mainly vegetarian diet[2]**									
**Girls**	-0.03	-0.32	0.27	-0.03	-0.32	0.27	0.03	-0.28	0.34
**Boys**	0.33	0.05	0.61	0.32	0.04	0.60	0.31	0.04	0.59
**Wave 3 (Girls n =679, Boys n =715)**									
**β estimate**	β	95%-CI		β	95%-CI		β	95%-CI	
**Total iron intake [mg/d][1]**									
**Girls**	0	-0.02	0.03	0	-0.02	0.03	0.01	-0.05	0.07
**Boys**	0	-0.02	0.02	0	-0.02	0.02	-0.06	-0.12	0.01
**Mainly vegetarian diet[2]**									
**Girls**	0.03	-0.08	0.15	0.03	-0.08	0.15	0.04	-0.08	0.16
**Boys**	0.10	-0.02	0.21	0.10	-0.02	0.21	0.05	-0.07	0.18
**Longitudinal (Girls n=402, Boys n=410)**									
**β estimate**	β	95%-CI		β	95%-CI		β	95%-CI	
**Total iron intake [mg/d][1]**									
**Girls**	0	-0.02	0.02	0	-0.03	0.02	0.05	-0.02	0.11
**Boys**	0	-0.02	0.03	0	-0.02	0.03	-0.03	-0.11	0.04
**Mainly vegetarian diet[2]**									
**Girls**	0.05	-0.07	0.18	0.05	-0.07	0.18	0.09	-0.05	0.22
**Boys**	0.05	-0.08	0.18	0.05	-0.08	0.18	0.02	-0.12	0.16

Adjusted for usual energy intake, age, BMI-z-score, sex, country and ISCED

[1]Usual dietary intakes of total iron, iron from heme sources and vitamin C were estimated from 24h dietary recalls linked to food composition tables.

[2]Children were classified as mainly vegetarian if they replied to the specific question to typically exclude meat, poultry, sausage and fish from their diet in the Food Frequency Questionnaire. Some of them however reported later on to occasionally consume meat or fish.

Abbreviations: β, β estimate; IDEFICS: Identification and Prevention of Dietary- and Lifestyle-Induced Health Effects in Children and Infants; W0, first examination wave; W3, third examination wave.

Table 10: Cross-sectional associations of usual total iron intake with serum ferritin [µg/L] and sensitivity analyses excluding children with high CRP >5 mg/L in the IDEFICS/I.Family study

Model	Main model			Sensitivity analysis including usual vitamin C intake[2]			Sensitivity analysis with usual iron intake from heme sources[2] as exposure		
**Wave 0 (2007/2008) (N=829)**									
Logistic Regression									
**Odds ratio for having a sufficient iron status (ref.: deficient status)[1]**	OR	95%-CI		OR	95%-CI		OR	95%-CI	
**Total iron intake [mg/day][2]**	1.43	0.95	2.20	1.42	0.91	2.28	1.27	0.76	2.11
**Mainly vegetarian diet[3] (ref: omnivore diet)**	0.48	0.08	9.20	0.48	0.08	9.21	0.51	0.08	9.85
**Vitamin C intake [mg/day][2]**				1.00	0.98	1.03			
Linear Model									
**β estimate**	β	95%-CI		β	95%-CI		β	95%-CI	
**Total iron intake [mg/day][2]**	1.12	-0.52	2.76	0.78	-1.00	2.57	2.54	0.10	4.97
**Mainly vegetarian diet[3]**	-2.62	-13.21	7.97	-2.46	-13.05	8.14	-1.25	-11.92	9.41
**Vitamin C intake [mg/day][2]**				0.04	-0.05	0.14			
**Wave 3 (2013/2014) (N=1,308)**									
Logistic Regression									
**Odds ratio for having a sufficient iron status (ref.: deficient status)[1]**	OR	95%-CI		OR	95%-CI		OR	95%-CI	
**Total iron intake [mg/day][2]**	1.09	0.92	1.31	1.09	0.91	1.32	1.84	1.08	3.24
**Mainly vegetarian diet[3] (ref: omnivore diet)**	0.38	0.20	0.78	0.38	0.20	0.78	0.60	0.28	1.34
**Vitamin C intake [mg/day][2]**				1.00	0.99	1.02			
Linear Model									
**β estimate**	β	95%-CI		β	95%-CI		β	95%-CI	
**Total iron intake [mg/day][2]**	0.48	-0.36	1.32	0.20	-0.69	1.10	3.80	1.44	6.16
**Mainly vegetarian diet[3] (ref: omnivore diet)**	-3.82	-8.17	0.52	-3.77	-8.11	0.57	-0.82	-5.53	3.89
**Vitamin C intake [mg/day][2]**				0.07	0	0.14			

Adjusted for usual energy intake, age, BMI-z-score, sex, country and ISCED

[1]Iron deficiency defined according to the WHO: children aged <5 years: serum ferritin <12 µg/L; children of 5 years or older: serum ferritin <15 µg/L

[2]Usual dietary intakes of total iron, iron from heme sources and vitamin C were estimated from 24h dietary recalls linked to food composition tables.

[3]Children were classified as mainly vegetarian if they replied to the specific question to typically exclude meat, poultry, sausage and fish from their diet in the Food Frequency Questionnaire. Some of them however reported later on to occasionally consume meat or fish.

Abbreviations: β, β estimate; IDEFICS: Identification and Prevention of Dietary- and Lifestyle-Induced Health Effects in Children and Infants; OR, Odds ratio.

Table 11: Longitudinal associations of usual total iron intake with serum ferritin [µg/L] at W3 (2013/2014) adjusted for serum ferritin at W0 (2007/08) and sensitivity analyses excluding children with high CRP >5 mg/L in the IDEFICS/I.Family study

**Model**	**Main model**			**Sensitivity analysis including usual vitamin C intake[2]**			**Sensitivity analysis with usual iron intake from heme sources[2] as exposure**		
**N=766**									
Logistic Regression									
**Odds ratio for having a sufficient iron status at W3 (ref.: deficient status)[1]**	OR	95%-CI		OR	95%-CI		OR	95%-CI	
**Total iron intake [mg/day][2]**	1.07	0.86	1.36	1.09	0.86	1.41	1.89	0.91	4.19
**Mainly vegetarian diet[3] (ref: omnivore diet)**	0.34	0.14	0.89	0.34	0.14	0.89	0.54	0.20	1.64
**Vitamin C intake [mg/day][2]**				1.00	0.98	1.02			
Linear Model									
**β estimate**	β	95%-CI		β	95%-CI		β	95%-CI	
**Total iron intake [mg/day][2]**	-0.08	-0.97	0.81	-0.16	-1.12	0.81	2.52	-0.09	5.13
**Mainly vegetarian diet[3] (ref: omnivore diet)**	-4.85	-9.92	0.21	-4.85	-9.92	0.22	-2.81	-8.29	2.68
**Vitamin C intake [mg/day][2]**				0.02	-0.07	0.10			

Adjusted for usual energy intake, age, BMI-z-score, sex, country, ISCED (all at W3) and ferritin at W0

[1]Iron deficiency defined according to the WHO: children aged <5 years: serum ferritin <12 µg/L; children of 5 years or older: serum ferritin <15 µg/L.

[2]Usual dietary intakes of total iron, iron from heme sources and vitamin C were estimated from 24h dietary recalls linked to food composition tables.

[3]Children were classified as mainly vegetarian if they replied to the specific question to typically exclude meat, poultry, sausage and fish from their diet in the Food Frequency Questionnaire. Some of them however reported later on to occasionally consume meat or fish.

Abbreviations: β, β estimate; IDEFICS: Identification and Prevention of Dietary- and Lifestyle-Induced Health Effects in Children and Infants; OR, Odds ratio; W0, first examination wave; W3, third examination wave.

Table 12: Cross-sectional and longitudinal associations (linear model) from W0 (2007/2008) to W3 (2013/2014) of usual total iron intake with serum transferrin [g/L] and sensitivity analyses excluding children with high CRP >5 mg/L in the IDEFICS/I.Family study

Model	Main model			Sensitivity analysis including usual vitamin C intake[1]			Sensitivity analysis with usual iron intake from heme sources[1] as exposure		
**Wave 0 (2007/2008)** (N=829)									
**β estimate**	β	95%-CI		β	95%-CI		β	95%-CI	
**Total iron intake[1] [mg/day]**	0	-0.03	0.03	0.01	-0.02	0.05	-0.01	-0.05	0.04
**Mainly vegetarian diet[2] (ref: omnivore diet)**	0.14	-0.06	0.34	0.14	-0.06	0.34	0.14	-0.06	0.34
**Wave 3 (2013/2014)** (N=1.308)									
**β estimate**	β	95%-CI		β	95%-CI		β	95%-CI	
**Total iron intake[1] [mg/day]**	0	-0.01	0.02	0	-0.02	0.02	0	-0.05	0.04
**Mainly vegetarian diet[2] (ref: omnivore diet)**	**0.08**	**0**	**0.16**	**0.08**	**0**	**0.16**	0.08	-0.01	0.17
**Longitudinal (N=768)**									
**β estimate**	β	95%-CI		β	95%-CI		β	95%-CI	
**Total iron intake[1] [mg/day]**	0	-0.01	0.02	0	-0.02	0.02	0.02	-0.03	0.07
**Mainly vegetarian diet[2] (ref: omnivore diet)**	0.07	-0.02	0.16	0.07	-0.03	0.16	0.09	-0.01	0.19

Adjusted for usual energy intake, age, BMI-z-score, sex, country and ISCED

[1]Usual dietary intakes of total iron, iron from heme sources and vitamin C were estimated from 24h dietary recalls linked to food composition tables.

[2]Children were classified as mainly vegetarian if they replied to the specific question to typically exclude meat, poultry, sausage and fish from their diet in the Food Frequency Questionnaire. Some of them however reported later on to occasionally consume meat or fish.

Abbreviations: β, β estimate; IDEFICS: Identification and Prevention of Dietary- and Lifestyle-Induced Health Effects in Children and Infants; W0, first examination wave; W3, third examination wave.

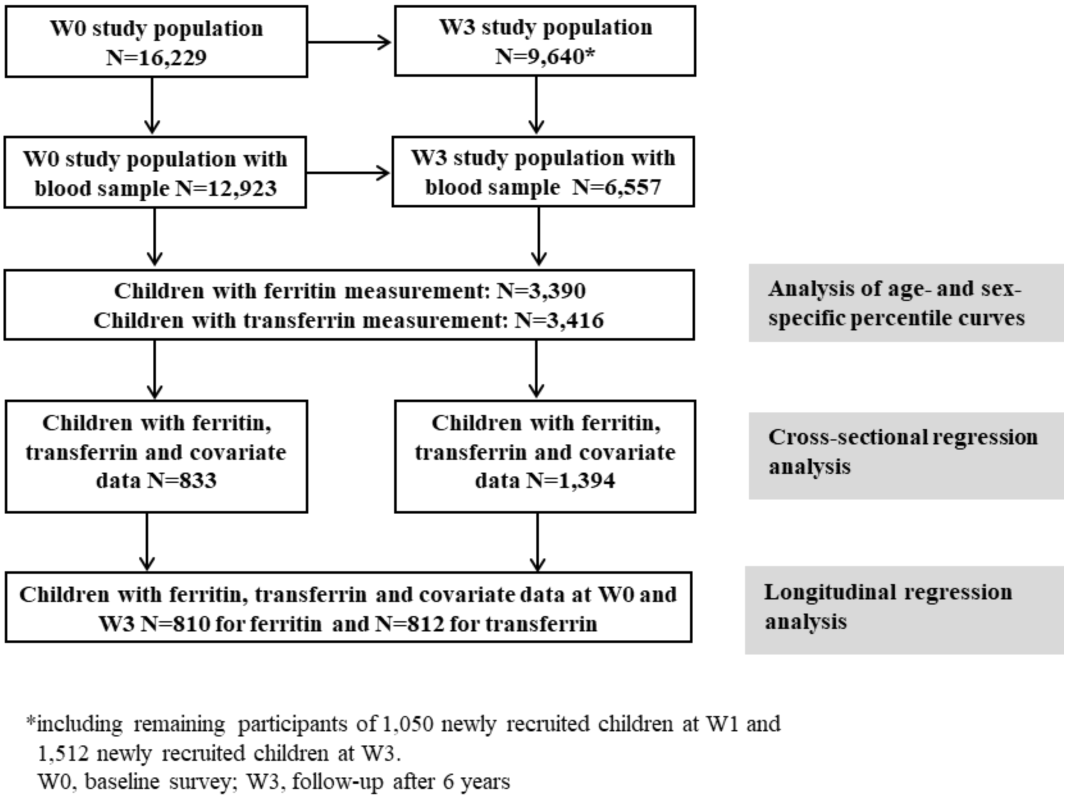

Supplementary Figure 1. Flow chart of inclusion of children of the IDEFICS/I.Family study into the analytical study samples. IDEFICS: Identification and Prevention of Dietary- and Lifestyle-Induced Health Effects in Children and Infants.

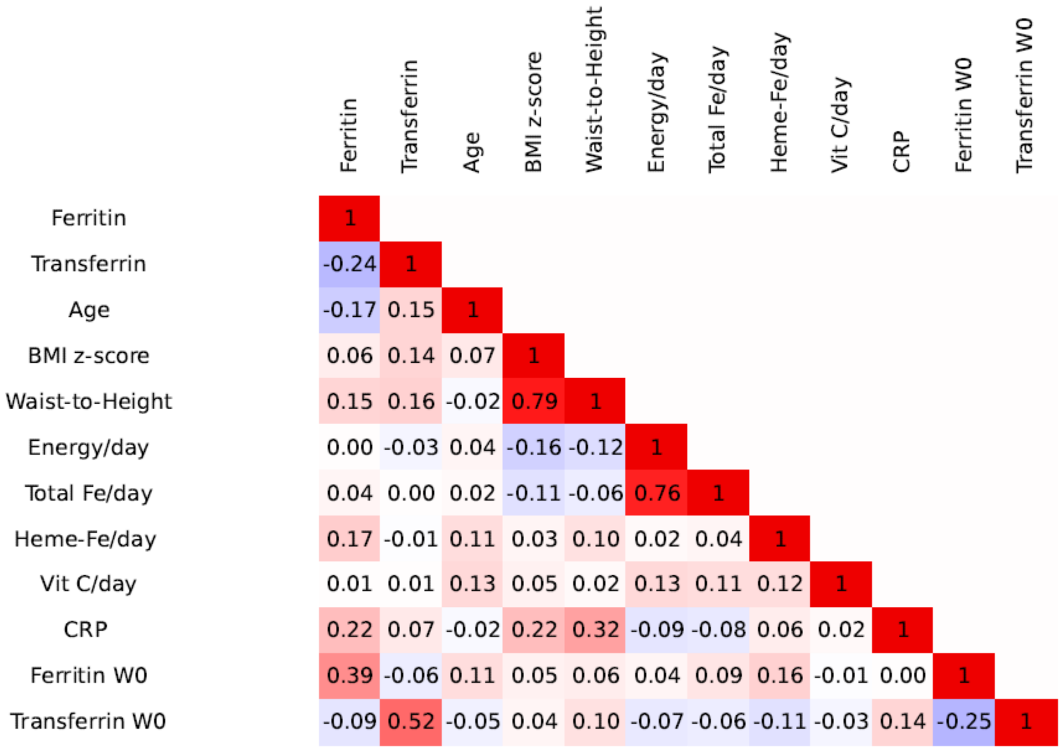

Supplementary Figure 2. Correlation coefficients of serum ferritin and transferrin concentrations with further dietary and metabolic variables in European children from the IDEFICS/I.Family study at W3. All variables presented in the figure were assessed at W3 except specified otherwise.

Total energy intake per day and dietary intakes of iron and vitamin C were estimated from 24h dietary recalls linked to food composition tables.

Abbreviations: CRP, C-reactive protein, Total Fe/day, total usual dietary iron intake per day; ; heme-Fe/day, usual iron intake from heme sources per day; VitC/day, usual vitamin C intake per day; IDEFICS: Identification and Prevention of Dietary- and Lifestyle-Induced Health Effects in Children and Infants; W0, first examination wave 2007-2008; W3, third examination wave 2013-2014.
